# Targeting mechanosensitive endothelial TXNDC5 to stabilize eNOS and reduce atherosclerosis in vivo

**DOI:** 10.1126/sciadv.abl8096

**Published:** 2022-01-21

**Authors:** Chih-Fan Yeh, Shih-Hsin Cheng, Yu-Shan Lin, Tzu-Pin Shentu, Ru-Ting Huang, Jiayu Zhu, Yen-Ting Chen, Sandeep Kumar, Mao-Shin Lin, Hsien-Li Kao, Po-Hsun Huang, Esther Roselló-Sastre, Francisca Garcia, Hanjoong Jo, Yun Fang, Kai-Chien Yang

**Affiliations:** 1 Division of Cardiology, Department of Internal Medicine and Cardiovascular Center, National Taiwan University Hospital, Taipei, Taiwan.; 2 Department and Graduate Institute of Pharmacology, National Taiwan University College of Medicine, Taipei, Taiwan.; 3 Department of Medicine, Biological Sciences Division and College, The University of Chicago, Chicago, IL, USA.; 4 Wallace H. Coulter Department of Biomedical Engineering, Georgia Institute of Technology and Emory University, Atlanta, GA, USA.; 5 Division of Cardiology, Department of Internal Medicine, Veteran General Hospital, Taipei, Taiwan.; 6 Department of Anatomic Pathology, Hospital General Universitario de Castellón, Castellón, Spain.; 7 Department of Vascular Surgery, Hospital General Universitario de Castellón, Castellón, Spain.; 8 Department of Health Sciences, Universidad CEU Cardenal Herrera, Valencia, Spain.; 9 Research Center for Developmental Biology and Regenerative Medicine, National Taiwan University, Taipei, Taiwan.; 10 Institute of Biomedical Sciences, Academia Sinica, Taipei, Taiwan.

## Abstract

Although atherosclerosis preferentially develops at arterial curvatures and bifurcations where disturbed flow (DF) activates endothelium, therapies targeting flow-dependent mechanosensing pathways in the vasculature are unavailable. Here, we provided experimental evidence demonstrating a previously unidentified causal role of DF-induced endothelial TXNDC5 (thioredoxin domain containing 5) in atherosclerosis. TXNDC5 was increased in human and mouse atherosclerotic lesions and induced in endothelium subjected to DF. Endothelium-specific *Txndc5* deletion markedly reduced atherosclerosis in *ApoE*^−/−^ mice. Mechanistically, DF-induced TXNDC5 increases proteasome-mediated degradation of heat shock factor 1, leading to reduced heat shock protein 90 and accelerated eNOS (endothelial nitric oxide synthase) protein degradation. Moreover, nanoparticles formulated to deliver *Txndc5*-targeting CRISPR-Cas9 plasmids driven by an endothelium-specific promoter (*CDH5*) significantly increase eNOS protein and reduce atherosclerosis in *ApoE*^−/−^ mice. These results delineate a new molecular paradigm that DF-induced endothelial TXNDC5 promotes atherosclerosis and establish a proof of concept of targeting endothelial mechanosensitive pathways in vivo against atherosclerosis.

## Teaser

Targeting flow-sensitive TXNDC5, an ER protein driving endothelial dysfunction by destabilizing eNOS, lessens atherosclerosis.

## INTRODUCTION

Atherosclerosis is a major cause of human morbidity and mortality. Although atherosclerotic lesions largely occur in specific arterial regions where disturbed blood flow (DF) activates endothelial cells (ECs) via mechanotransduction (*1*–*4*), current therapies for atherosclerosis mainly target systemic risk factors (e.g., hypercholesterolemia and hypertension) rather than the vasculature per se and remain suboptimal. This underscores the significance to identify new atherosclerosis-causing endothelial mechanosensitive mechanisms and, moreover, to develop novel therapeutic approaches to target the vascular wall. Atherosclerotic lesions develop preferentially at arterial sites of curvature, branching, and bifurcation, where ECs are exposed to multidirectional DF featuring oscillation, flow reversal, and low time-averaged shear stress (*1*–*3*). In contrast, ECs in the straight parts of the arteries are exposed to unidirectional flow (UF) and are largely resistant to atherosclerosis. Hemodynamic forces are major regulators of endothelial health and disease. DF stimulates low-grade inflammation, compromises vascular integrity, and elevates glycolysis in atherosusceptible endothelium, while UF promotes a quiescent endothelial phenotype resistant to atherogenesis (*3*, *5*–*7*). DF-induced endothelial activation contributes to a wide range of vascular diseases such as aneurysms, arteriovenous malfunctions, and atherosclerosis (*1*–*3*). A major molecular signature of DF-activated, atherosusceptible endothelial phenotype is decreased expression of endothelial nitric oxide synthase [eNOS; encoded by nitric oxide synthase 3 (*NOS3*)] (*8*). In vivo and in vitro studies have shown that *NOS3* expression is sustained in ECs under UF but significantly reduced in response to DF. *NOS3* is transcriptionally down-regulated in DF-exposed endothelium (*9*, *10*). Protein (de)stability of eNOS as a function of hemodynamics, however, remains poorly understood.

Exploiting an RNA sequencing (RNA-seq) dataset acquired from human aortic endothelial cells (HAEC) exposed to UF or DF (*11*), generated by an in vitro dynamic flow device that accurately reproduced arterial flow waveforms measured in humans (*12*), we identified thioredoxin domain containing 5 (TXNDC5) as a potential novel mechanosensitive molecule contributing to endothelial responses to atherorelevant hemodynamics. TXNDC5, an endoplasmic reticulum (ER) protein with the enzyme activity of protein disulfide isomerase (PDI), is highly expressed in vascular endothelium and therefore also known as endothelial PDI (Endo-PDI) (*13*). In addition, TXNDC5 is induced by hypoxia in ECs and mediates tumor necrosis factor–α (TNFα)–induced angiogenesis (*13*, *14*). We have recently demonstrated that TXNDC5 drives fibroblast activation and contributes to cardiac, pulmonary, and renal fibrosis (*15*–*17*). Nevertheless, the role of TXNDC5 in endothelial mechanotransduction and atherogenesis remains completely unknown.

Here, we reported a critical yet previously unrecognized role of TXNDC5 in DF-induced endothelial activation and atherosclerosis. Endothelial TXNDC5 is significantly increased in human atherosclerotic lesions and up-regulated by DF in vivo and in vitro. We engineered global and endothelium-specific *Txndc5* knockout mouse lines showing that *Txndc5* deletion, particularly in vascular endothelium, causatively reduces atherosclerosis in vivo. We further delineated novel molecular mechanisms by which DF induces endothelial TXNDC5 expression, leading to destabilized eNOS protein, endothelial activation, and atherogenesis. Capitalizing on this new mechanistic insight, we devised a targeted nanomedicine platform combining nanoparticles, an endothelium-specific promoter, and the CRISPR-Cas9 (CRISPR-associated protein 9) technology to specifically delete *Txndc5* in vascular endothelium in vivo, which significantly reduced atherosclerosis in *ApoE^−/−^* mice.

## RESULTS

### TXNDC5 is induced in the endothelium exposed to DF and up-regulated in atherosclerotic lesions

**Fig. 1.**
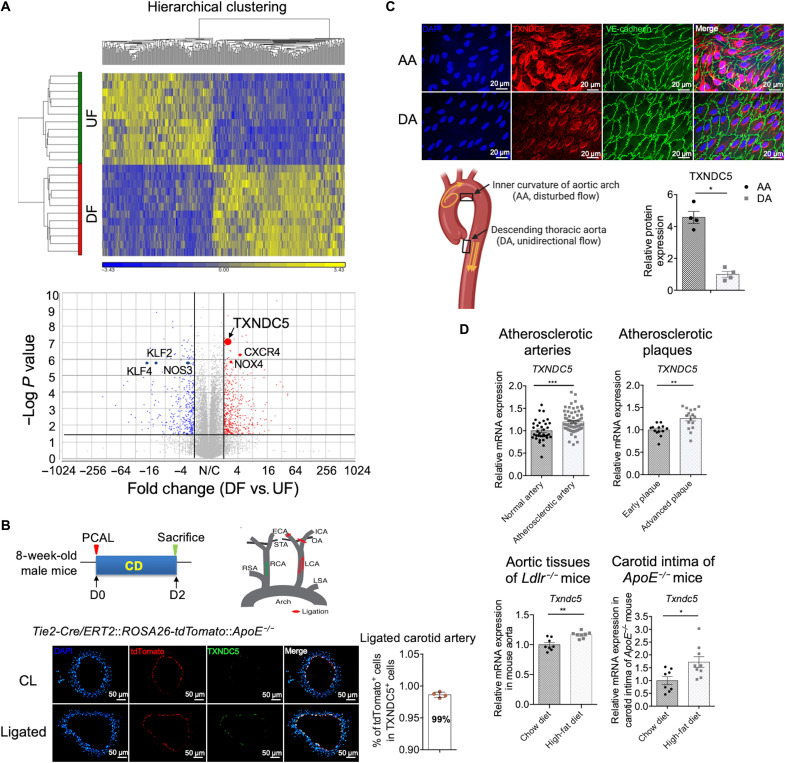
TXNDC5 is induced in the endothelium exposed to DF and up-regulated in atherosclerotic lesions. (**A**) RNA-seq data performed on HAEC exposed to UF or DF generated using an in vitro dynamic flow device for 24 hours were reanalyzed. The heatmap and unsupervised hierarchical clustering of the RNA-seq data showed a clear demarcation of between DF- and UF-exposed HAEC. *TXNDC5* (fold change of 2.43, *P* = 7.2 × 10^−8^) and atherogenic factors including *CXCR4* and *NOX4* were significantly up-regulated, whereas multiple atheroprotective factors such as *KLF2*, *KLF4*, and *NOS3* were down-regulated, in HAEC exposed to DF. (**B**) Partial carotid ligation (PCAL; illustrated in the scheme) was performed to induce DF, which activates ECs and promotes atherosclerosis, in the left carotid artery (LCA) of the tamoxifen-injected *Tie2-Cre/ERT2::ROSA26-tdTomato::ApoE^−/−^* mice, in which tdTomato (red) was induced in cells expressing an endothelium-enriched *Tie2-Cre* recombinase. Immunofluorescence (IF) staining of ligated LCA and nonligated contralateral carotid artery (CL) 2 days after the PCAL + chow diet (CD) showed that TXNDC5 expression (green) was significantly induced in ligated LCA exposed to DF. Almost all (~99%) of TXNDC5-positive cells were tdTomato positive (*n* = 4). (**C**) En face staining of the mouse aorta showed increased TXNDC5 expression in the endothelium of aortic arch (AA; inner curvature) compared to that of descending thoracic aorta (DA) in C57BL/6 mice (*n* = 4). (**D**) Reanalysis of microarray datasets obtained from human atherosclerotic arteries (GSE100927), plaques (GSE28829), and aortic tissues of *Ldlr^−/−^* mice (GSE69187) revealed that *TXNDC5* was significantly up-regulated in human atherosclerotic arteries (compared to healthy arteries) and in advanced atherosclerotic plaques (compared to early plaques), as well as in the aorta from high-fat diet (HFD)–fed (compared to CD-fed) *Ldlr^−/−^* mice. *Txndc5* mRNA level was more robustly up-regulated in the carotid intima of HFD-fed, compared to CD-fed, *ApoE^−/−^* mice. (*P < 0.05, **P < 0.01, and ***P < 0.001 determined using two-tailed Mann-Whitney *U* test).

To identify novel molecular determinants underlying the mechanoregulation of endothelium by atherorelevant flows, we analyzed the RNA-seq data acquired from HAEC exposed to 24 hours of dynamic flows (*11*) generated by an in vitro flow device that accurately recreates DF measured in human carotid sinus or UF measured in human distal carotid artery (*12*). The heatmap and hierarchical clustering analyses of the RNA-seq results showed a clear demarcation between DF- and UF-exposed HAEC (Fig. 1A, top). Consistent with atherogenic response induced by DF, several atheroprotective factors such as Kruppel-like factor 2 (*KLF2*), *KLF4*, and *NOS3* were down-regulated, whereas atherogenic factors including C-X-C motif chemokine receptor 4 (*CXCR4*) and NADPH oxidase 4 (*NOX4*) were up-regulated in HAEC exposed to DF, compared to cells exposed to UF (volcano plot in Fig. 1A, bottom). Endothelial *TXNDC5* was one of the most significantly up-regulated (fold change of 2.43, *P* = 7.2 × 10^−8^) mechanoresponsive genes induced by DF. Although TXNDC5 is highly expressed in vascular endothelium and therefore also known as Endo-PDI (*13*), it has never been investigated in the context of endothelial dysfunction or atherosclerosis. Consistent with RNA-seq results, quantitative real-time polymerase chain reaction (qRT-PCR; fig. S1A) and immunoblotting (fig. S1B) validated a robust up-regulation of TXNDC5 transcripts and proteins in HAEC by DF. DF-induced up-regulation of *TXNDC5* was accompanied by reduced expression of flow-sensitive atheroprotective genes and proteins, including *NOS3*/eNOS and *KLF2*, validated by qRT-PCR (fig. S1, A and B).

Next, partial carotid artery ligation (PCAL; Fig. 1B), an established in vivo model to induce acute DF with characteristics of low and oscillatory wall shear stress in the common carotid artery (*18*), was performed in a transgenic reporter mouse line in the *ApoE^−/−^* background (*Tie2-Cre/ERT2*::*ROSA26-tdTomato*::*ApoE^−/−^*) that allows visualization of the endothelium with tdTomato following tamoxifen induction. Immunofluorescence (IF) staining of the mouse carotid artery sections showed that TXNDC5 expression was significantly increased in the ligated left carotid artery (LCA), compared to nonligated contralateral carotid artery (CL), 2 days after the PCAL. Notably, TXNDC5 was largely localized (>99%) in tdTomato-positive ECs, indicating its specific enrichment in the arterial endothelium (Fig. 1B). Consistent with the DF-induced TXNDC5 up-regulation observed in the carotid endothelium, en face IF staining of the mouse aortic arch (AA; inner curvature) and descending thoracic aorta (DA), two arterial regions exposed to chronic DF and UF in intact animals, respectively, revealed that TXNDC5 was up-regulated, whereas eNOS was down-regulated, in the endothelium of DF-exposed AA, compared to that in UF-exposed DA (Fig. 1C and fig. S1C). Together, these results demonstrate the flow-sensitive nature of the endothelium-enriched protein TXNDC5.

To determine a possible role of TXNDC5 in atherosclerosis, we analyzed the expression level of TXNDC5 in the atherosclerotic arterial tissues from both humans and mice. Reanalysis of multiple microarray datasets obtained from human arterial tissues (GSE100927) and plaques (GSE28829) showed that *TXNDC5* expression was significantly up-regulated in human atherosclerotic arteries (compared to healthy arteries) and in advanced atherosclerotic plaques (compared to early plaques), as well as in the aorta from high-fat diet (HFD)–fed *Ldlr^−/−^* mice [GSE69187; compared to those from chow diet (CD)–fed *Ldlr^−/−^* mice] (Fig. 1D). Furthermore, *Txndc5* mRNA expression was also robustly up-regulated in the carotid intima of HFD-fed, compared to CD-fed, *ApoE^−/−^* mice (Fig. 1D). These data collectively provided the first line of evidence showing an increased TXNDC5 expression in atherosclerotic lesions.

### Global and endothelium-specific deletion of *Txndc5* significantly reduces atherosclerosis in vivo

**Fig. 2.**
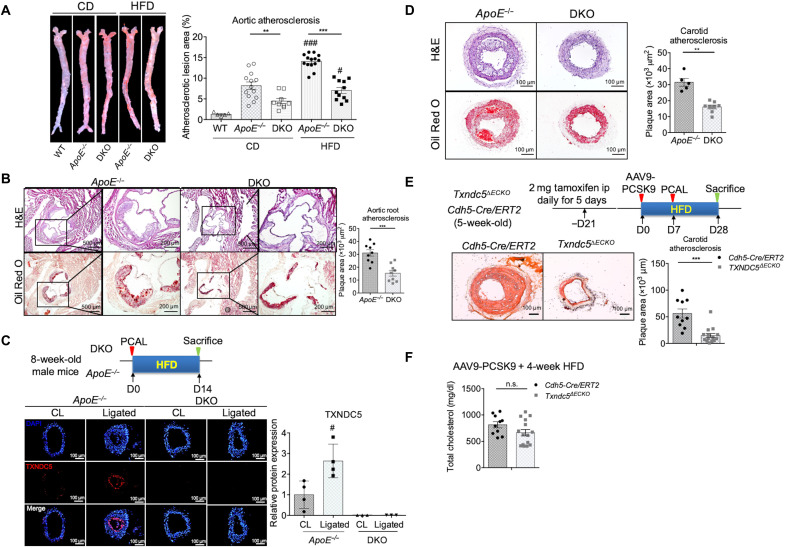
Global and endothelial-specific deletion of DF-induced *Txndc5* significantly reduces atherosclerosis in vivo. (**A**) Global *Txndc5* deletion in male *ApoE^−/−^* mice [*Txndc5^−/−^*::*ApoE^−/−^* (DKO)] significantly reduced aortic atherosclerotic burden (en face Oil Red O–stained area) under 12-week CD or HFD (see text) starting from 8 weeks old (*n* = 5 to 14). WT, wild type. (**B**) Atherosclerotic lesions at aortic sinus were markedly reduced by global *Txndc5* deletion in DKO mice compared to *ApoE^−/−^* under 12-week HFD (*n* = 9). H&E, hematoxylin and eosin. (**C**) IF staining of ligated LCA from male *ApoE^−/−^* mice (2 weeks after PCAL and HFD) showed marked up-regulation of TXNDC5 in the intima of LCA. No DF-induced TXNDC5 was detected in DKO mice (*n* = 3 to 4). (**D**) Global *Txndc5* deletion significantly reduced DF-induced atherosclerosis in the ligated LCA in DKO mice compared to *ApoE^−/−^* mice (*n* = 5 to 8). (**E**) Endothelial-specific deletion of *Txndc5* by *Cdh5-Cre* recombinase significantly reduced DF-induced atherosclerosis in the ligated LCA in hypercholesterolemic *Cdh5-Cre/ERT2::Txndc5^fl/fl^* (*Txndc5*^∆*ECKO*^) mice (seven males and eight females) compared to tamoxifen-treated *Cdh5-Cre/ERT2* controls (six males and four female). Hypercholesterolemia was induced by PCSK9 overexpression (one tail vein injection of AAV9-PCSK9; 1 × 10^11^ VG) and 4-week HFD (*n* = 10 to 15). ip, intraperitoneally. (**F**) No significant differences in total plasma cholesterol levels were observed in *Txndc5*^∆*ECKO*^ and *Cdh5-Cre/ERT2* mice subjected to AAV9-PCSK9 injection and fed with 4-week HFD (*n* = 10 to 15) (**P < 0.01, ***P < 0.001, ^#^*P* < 0.05, and ^###^*P* < 0.001 between treatments of the same genotype; n.s., nonsignificant using two-tailed Mann-Whitney *U* test).

To determine whether TXNDC5 causatively drives atherosclerosis in vivo, we then engineered a new mouse line [*Txndc5^−/−^*::*ApoE^−/−^* (DKO)] in which *Txndc5* is globally deleted in the background of *ApoE^−/−^* mice. Plaque formation at the aorta and aortic sinuses was assessed in DKO and *ApoE^−/−^* mice following 12 weeks of HFD. As shown in Fig. 2A, atherosclerotic burden (en face Oil Red O–stained area) was significantly reduced in the aorta from DKO, compared to that from *ApoE^−/−^,* mice. The atherosclerotic lesions in the aortic sinuses were also reduced markedly (by ~50%) in DKO, compared to *ApoE^−/−^*, mice (Fig. 2B) without affecting the plasma total cholesterol and triglyceride levels (fig. S2A). In addition, plasma levels of inflammatory markers, including interleukin-1β (IL-1β) and C-reactive protein (CRP), were indistinguishable between *ApoE^−/−^* and DKO mice, suggesting that *Txndc5* deletion has no effects on systemic inflammation during atherogenesis (fig. S2B). IF staining of the ligated LCA from *ApoE^−/−^* mice 2 weeks following PCAL and HFD showed marked up-regulation of TXNDC5 in the carotid intima (Fig. 2C), accompanied by the development of substantial carotid arterial plaques (Fig. 2D). The size of the DF-induced carotid plaques following PCAL/HFD was significantly reduced in DKO compared to *ApoE^−/−^* mice (Fig. 2D).

To further determine the causal role of DF-induced endothelial TXNDC5 in atherosclerosis, we generated a mouse line [*Cdh5-Cre/ERT2::Txndc5^fl/fl^* (*Txndc5*^∆*ECKO*^)] in which *Txndc5* can be deleted in adult vascular endothelium by a tamoxifen-induced Cre recombinase driven by the endothelium-specific *Cdh5* (*VE-Cadherin*) promoter (*19*). The efficiency of *Txndc5* depletion in the endothelium of *Txndc5*^∆*ECKO*^ was confirmed by qRT-PCR using RNA isolated from endothelium-enriched intima of CL (fig. S3A) and by IF staining of ligated LCA (fig. S3B). Three weeks after tamoxifen injection, *Txndc5*^∆*ECKO*^ and control mice (*Cdh5-Cre/ERT2*) were subjected to proprotein convertase subtilisin/kexin type 9 (PCSK9) overexpression using adeno-associated virus 9 (AAV9-PCSK9) followed by HFD and PCAL, an established model of accelerated carotid atherosclerosis within 3 to 4 weeks (experimental scheme shown in Fig. 2E) (*20*). The degree of hypercholesterolemia induced by AAV9-PCSK9 injection and 4-week HFD was similar in *Txndc5*^∆*ECKO*^ and control mice (Fig. 2F). Oil Red O staining showed considerable atherosclerotic lesions and intimal thickening in the ligated LCA from control mice following PCSK9 overexpression and PCAL; the extent of DF-induced atherosclerosis, by contrast, was significantly lower in *Txndc5*^∆*ECKO*^ mice with PCSK9 overexpression (Fig. 2E). Consistent with the observation made in the AAV9-PCSK9 mouse model, atherosclerotic lesions were markedly reduced in *Txndc5*^∆*ECKO*^, compared with control (*Cdh5-Cre/ERT2*), mice in the *ApoE^−/−^* background 2 weeks following PCAL and HFD (fig. S3C). Moreover, the atherosclerotic lesions in the aortic sinuses were markedly reduced in *Txndc5*^∆*ECKO*^, compared to control, mice under 3-month HFD following PCSK9 overexpression (fig. S3D), without affecting the plasma total cholesterol levels (fig. S3E). Together, these results demonstrate a casual and significant role of endothelial TXNDC5 in atherogenesis in vivo. Endothelial *Txndc5* deletion markedly reduces DF-induced atherosclerosis and plaque formation in hyperlipidemic mice.

### DF-induced TXNDC5 down-regulates eNOS protein in vascular endothelium

**Fig. 3.**
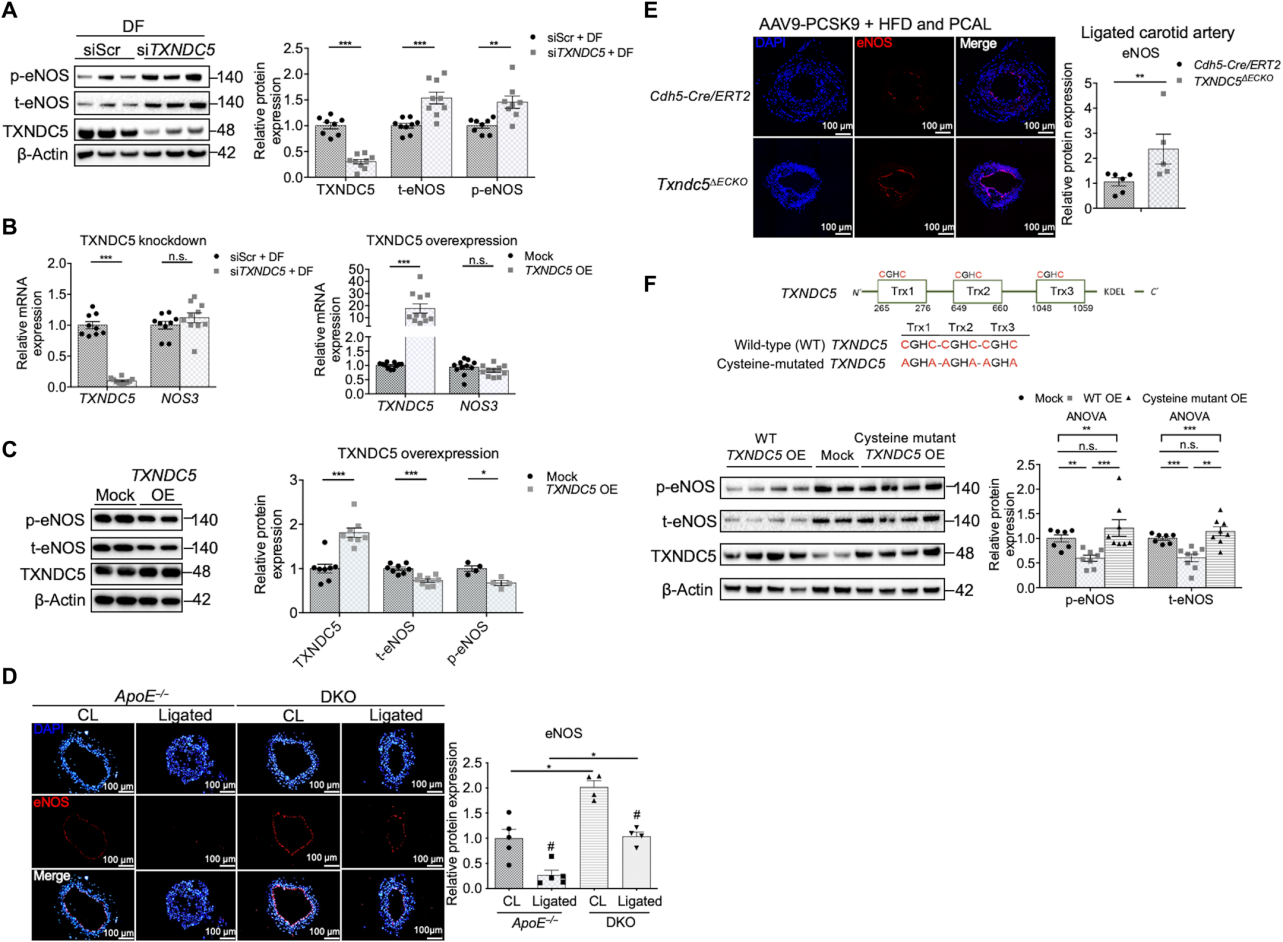
DF up-regulates endothelial TXNDC5 to increase protein degradation of eNOS. (**A**) In HAEC under DF, knockdown of *TXNDC5* with siRNA (si*TXNDC5*) significantly increased the expression levels of t-eNOS and p-eNOS (Ser^1177^). siScr, nontargeting scrambled control (*n* = 8 to 9). (**B**) *NOS3* (which encodes eNOS) mRNA was unaffected by either knockdown or overexpression (OE) of *TXNDC5* in HAEC (*n* = 9 to 11). (**C**) *TXNDC5* overexpression led to a marked down-regulation of p-eNOS and t-eNOS in HAEC (*n* = 4 to 8). (**D**) Global *Txndc5* deletion in *ApoE^−/−^* mice (DKO) led to marked up-regulation of eNOS (red; intensity measured along the vessel lumen) in the intima of nonligated CL and in ligated LCA compared to the counterparts in *ApoE^−/−^* mice (*n* = 3). (**E**) Endothelium-specific deletion of *Txndc5* by *Cdh5*-*Cre/ERT2* restored eNOS expression (intensity measured along the vessel lumen) in ligated LCA in *Txndc5*^∆*ECKO*^ mice subjected to AAV9-PCSK9 injection and 4-week HFD (*n* = 5 to 6). (**F**) Cysteine-to-alanine mutations that were introduced at all three thioredoxin domains of TXNDC5 (shown in the scheme), which abolish its PDI activity, eliminated the action of TXNDC5 to reduce eNOS proteins in HAEC (*n* = 7 to 8) (*P < 0.05, **P < 0.01, ***P < 0.001, and ^#^*P* < 0.05, between treatments of the same genotype determined using two-tailed Mann-Whitney *U* test or ANOVA test). ANOVA, analysis of variance.

Because eNOS plays a critical role in regulating flow-dependent vascular functions and suppressing atherogenesis (*8*, *21*, *22*), we determined whether TXNDC5, which is also flow dependent, has any effect on eNOS expression in the endothelium. In HAEC under DF, knockdown of *TXNDC5* with small interfering RNA (siRNA) (si*TXNDC5*) significantly increased the protein expression levels of total (t-eNOS) and phospho-eNOS (p-eNOS; Ser^1177^) (Fig. 3A), without affecting *NOS3* transcripts (Fig. 3B). In agreement with these results, *TXNDC5* overexpression in HAEC led to marked down-regulation of t-eNOS and p-eNOS protein, whereas the *NOS3* transcript level was not affected (Fig. 3, B and C). Consistent with these in vitro findings, IF staining detected a significantly higher eNOS expression level in both nonligated CL and ligated LCA in DKO, compared to those in *ApoE^−/−^*, mice (Fig. 3D). Endothelium-specific deletion of *Txndc5* also markedly increased eNOS expression in the ligated LCA in *Txndc5*^∆*ECKO*^, compared with that in control (*Cdh5-Cre/ERT2*), mice with hyperlipidemia induced by PCSK9 overexpression and HFD (Fig. 3E).

Nitric oxide (NO) production by eNOS plays a critical role in maintaining endothelial homeostasis and function. Consistent with the observed repressive effects of TXNDC5 on eNOS expression, *TXNDC5* knockdown significantly increased, whereas *TXNDC5* overexpression decreased, NO production in HAEC (fig. S4A). Because eNOS and NO are required for the maintenance of endothelial integrity (*23*), we next determined the impact of TXNDC5 on endothelial integrity using transendothelial electrical resistance (TEER) measurement in HAEC. In line with the observed inverse relationship between TXNDC5 and eNOS/NO levels in HAEC, knockdown of *TXNDC5* increased, while overexpression of *TXNDC5* reduced, TEER measured in HAEC (fig. S4B). *TXNDC5* knockdown–induced increase in TEER was blocked by the treatment with NOS inhibitor l-N^G^-nitroarginine methyl ester (l-NAME; 200 μM), whereas *TXNDC5* overexpression–mediated TEER reduction was mitigated by the treatment with a NO donor *S*-nitroso-*N*-acetyl-d, l-penicillamine (SNAP; 2 μM) in HAEC (fig. S4B). In addition, inhibition of eNOS activity in vivo by l-NAME (4.3 mM in drinking water) markedly abrogated the atheroprotective effects of *Txndc5* deletion in *ApoE^−/−^* mice following 2-week PCAL + HFD (fig. S4C). Collectively, these data suggest that excessive TXNDC5 disrupts endothelial function and integrity through reducing eNOS and NO production, thereby promoting atherogenesis.

To determine whether the PDI function of TXNDC5 is required for its capacity to reduce eNOS protein, we in vitro transcribed mRNAs from wild-type (WT) and cysteine-mutated *TXNDC5* constructs, where cysteines in all three thioredoxin domains were replaced by alanine to abolish its PDI activity (mutation scheme shown in Fig. 3F). Overexpression of WT, but not cysteine-mutated, *TXNDC5* mRNA reduced eNOS protein levels in HAEC, suggesting that TXNDC5 suppresses eNOS expression through, at least partially, its PDI activity (Fig. 3F). Together, these data suggest that endothelial TXNDC5 contributes to atherogenesis by posttranscriptional down-regulation of eNOS protein through its PDI activity.

### TXNDC5 destabilizes eNOS protein by reducing HSP90 in ECs

**Fig. 4.**
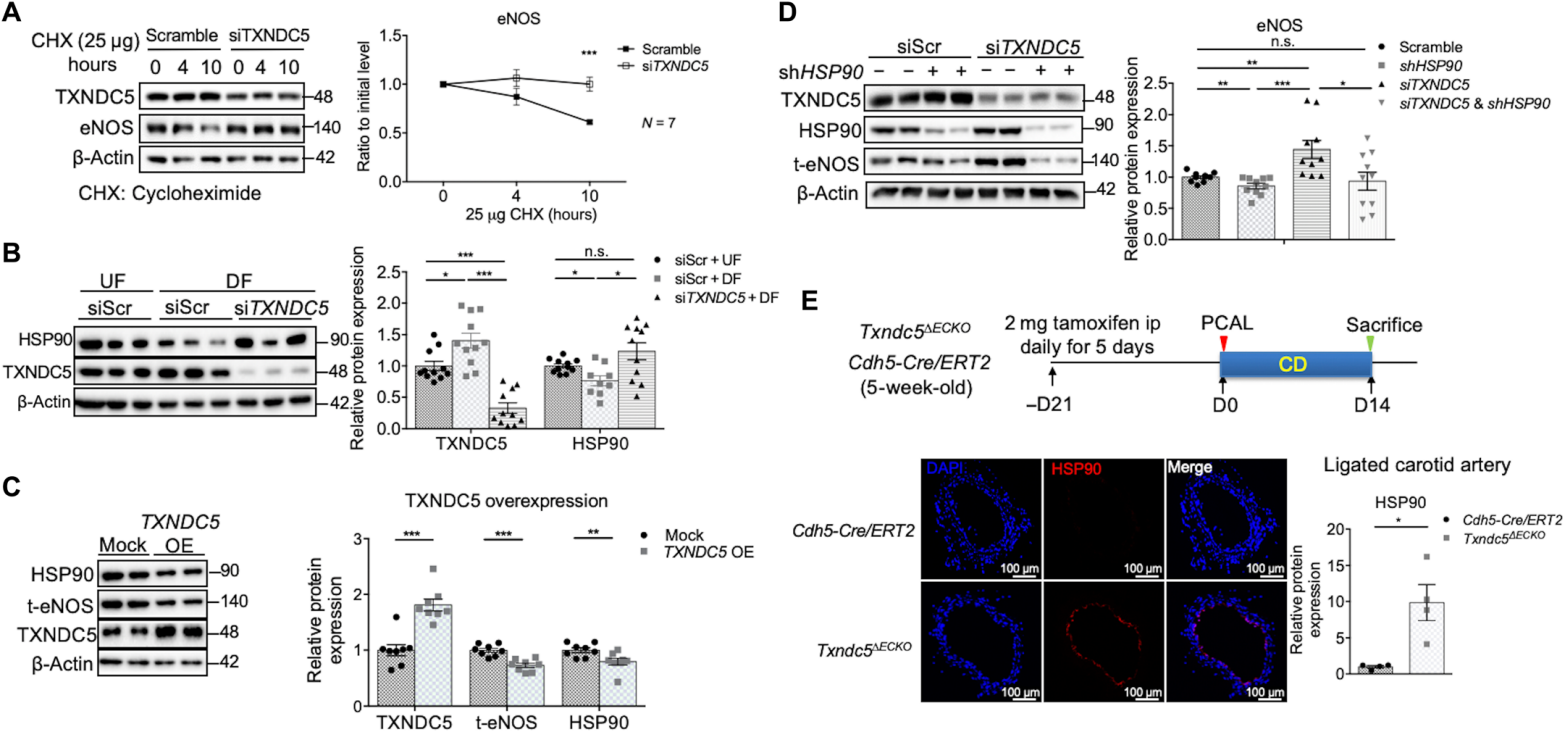
TXNDC5 down-regulates HSP90 to destabilize eNOS protein in ECs. (**A**) Cycloheximide (CHX; 25 μg/ml) protein chase assay demonstrated that *TXNDC5* knockdown decelerated eNOS protein degradation in HAEC (*n* = 7). (**B**) DF reduced HSP90 protein levels in HAEC, which was reversed by *TXNDC5* knockdown. siScr, nontargeting scrambled control (*n* = 9 to 11). (**C**) *TXNDC5* overexpression led to significant down-regulation of HSP90 and eNOS proteins in HAEC (*n* = 8). (**D**) Knocking down *HSP90* with short hairpin RNA (shRNA) abolished the increased eNOS protein in HAEC with *TXNDC5* knockdown (*n* = 10). (**E**) Endothelium-specific deletion of *Txndc5* by *Cdh5*-*Cre/ERT2* restored HSP90 expression (intensity measured along the vessel lumen) in the endothelium of ligated LCA in *Txndc5*^∆*ECKO*^ mice (*n* = 4). (*P < 0.05, **P < 0.01, and ***P < 0.001 determined using two-tailed Mann-Whitney *U* test).

To delineate how TXNDC5 mediates the posttranscriptional down-regulation of eNOS in ECs, a cycloheximide protein chase assay was performed to assess eNOS protein stability in response to *TXNDC5* depletion. As shown in Fig. 4A, *TXNDC5* knockdown in HAEC with siRNA (si*TXNDC5*) led to slower eNOS protein degradation, compared to cells treated with scramble siRNA, demonstrating that TXNDC5 destabilizes eNOS protein in the endothelium. Coimmunoprecipitation experiments, however, did not detect a physical binding between TXNDC5 and eNOS protein (fig. S5A), suggesting that TXNDC5-induced eNOS destabilization is not mediated through a direct interaction with eNOS.

To identify the potential molecular determinant(s) mediating TXNDC5-dependent posttranscriptional suppression of eNOS, RNA-seq was performed on HAEC with (si*TXNDC5*) and without (scramble siRNA) *TXNDC5* depletion. RNA-seq identified a total of 93 genes (71 up-regulated and 22 down-regulated) that were significantly differentially expressed (absolute fold change of ≥1.5, false discovery rate–corrected *P* < 0.05) with *TXNDC5* knockdown in HAEC (table S1). Gene ontology (GO) and pathway analyses of *TXNDC5*-regulated transcripts in ECs identified a significant enrichment of genes that are involved in heat shock response and, in particular, the regulation of HSF1 (heat shock factor 1)–mediated heat shock response (fig. S5, B and C).

Among the heat shock proteins that were induced by *TXNDC5* knockdown in HAEC, heat shock protein 90 (HSP90/*HSP90AA1*) has been reported as a key molecular chaperone to stabilize eNOS protein in the endothelium (*24*). We observed that HSP90 protein was significantly reduced by DF in HAEC (fig. S6A). We therefore hypothesized that DF-induced HSP90 down-regulation could mediate TXNDC5-dependent eNOS protein destabilization in the endothelium. Consistent with this hypothesis and in line with the RNA-seq results, DF-induced HSP90 down-regulation was significantly abrogated by *TXNDC5* knockdown (Fig. 4B). Moreover, *TXNDC5* overexpression led to a significant down-regulation of HSP90 (and eNOS) protein in HAEC (Fig. 4C). Notably, we showed that TXNDC5 negatively regulates HSP90 expression transcriptionally, as *TXNDC5* knockdown up-regulated, whereas *TXNDC5* overexpression down-regulated, *HSP90AA1* transcripts in HAEC (fig. S6B). Increased eNOS protein in *TXNDC5*-depleted HAEC was largely abrogated by knockdown (Fig. 4D) or pharmacological inhibition (with geldanamycin; fig. S6C) of HSP90, supporting that TXNDC5-mediated eNOS protein degradation was HSP90 dependent. In agreement with these in vitro findings, targeted deletion of endothelial *Txndc5* markedly increased eNOS (fig. S6D) and HSP90 (Fig. 4E) expression in the carotid endothelium in *Txndc5*^∆*ECKO*^, compared to control, mice following 2-week PCAL. These results collectively demonstrate that endothelial TXNDC5 destabilizes eNOS protein by transcriptional down-regulation of HSP90, a molecular chaperone essential to stabilize eNOS protein in the vascular endothelium.

### TXNDC5 suppresses *HSP90* expression via ubiquitin-dependent HSF1 degradation in the endothelium

**Fig. 5.**
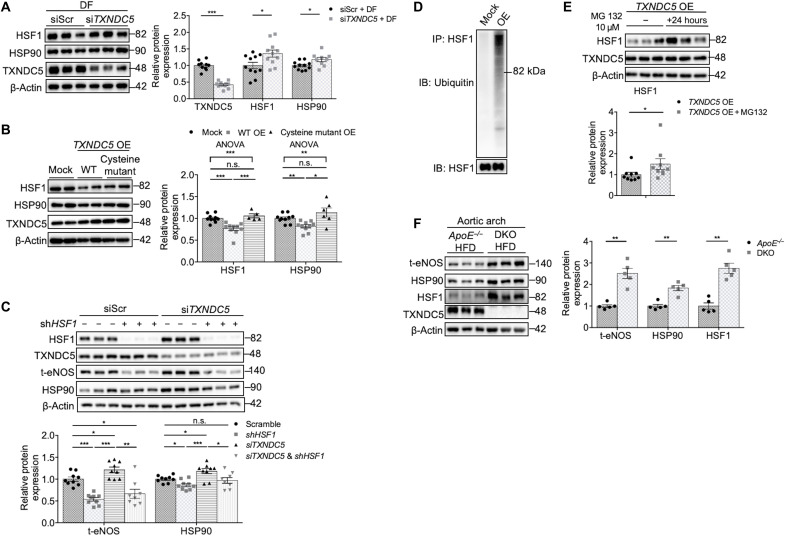
TXNDC5 increases ubiquitin-dependent HSF1 degradation to decrease HSP90 and eNOS in vascular endothelium. (**A** and **B**) Knocking down *TXNDC5* increased, whereas overexpression of *TXNDC5* reduced, HSF1 protein expression in HAEC. Overexpression of WT, but not cysteine-mutated, *TXNDC5* led to down-regulation of HSF1 and HSP90 proteins in HAEC. siScr, nontargeting scrambled control (*n* = 5 to 11). (**C**) Knocking down *HSF1* (with shRNA; sh*HSF1*) abrogated the up-regulation of HSP90 and eNOS in HAEC with *TXNDC5* knockdown (*n* = 8 to 9). (**D**) Immunoblots of HSF1 pulled-down protein lysates showed markedly increased ubiquitination of HSF1 in TXNDC5-overexpressed, compared to mock-treated, HAEC. (**E**) Treatment with a proteasome inhibitor MG132 (10 μM) abolished down-regulation of HSF1 in TXNDC5-overexressing HAEC (*n* = 9). (**F**) Immunoblotting showed marked up-regulation of t-eNOS, HSP90, and HSF1 protein levels in the AA from DKO, compared with *ApoE^−/−^*, mice fed with HFD (*n* = 5). (*P < 0.05, **P < 0.01, and ***P < 0.001 determined using two-tailed Mann-Whitney *U* test or ANOVA test).

Expression of HSP family genes, including *HSP90*, is primarily controlled by HSF1 in both vertebrates and invertebrates (*25*). The aforementioned RNA-seq analysis (fig. S5C) also suggests a potential involvement of HSF1-mediated heat shock response in TXNDC5-induced endothelial regulation. We therefore hypothesized that TXNDC5-mediated *HSP90* transcriptional down-regulation is dependent on HSF1, the role of which in endothelial mechanotransduction remains unknown. HSF1 protein, but not transcript, was markedly down-regulated in DF-exposed, compared to UF-exposed, HAEC (fig. S7, A and B). *TXNDC5* knockdown significantly increased HSF1 protein (Fig. 5A), without affecting *HSF1* transcript (fig. S7C), levels in DF-exposed HAEC, where reduced HSP90 protein was also restored (Figs. 4B and 5A). Moreover, overexpression of *TXNDC5* in HAEC significantly reduced HSF1 protein (Fig. 5B) but not its transcript levels (fig. S7C). Overexpression of cysteine-mutated *TXNDC5* did not affect the expression levels of HSF1 or HSP90 in HAEC (Fig. 5B), suggesting that TXNDC5-mediated HSF1/HSP90 regulation is dependent on the PDI activity of *TXNDC5*. *TXNDC5* depletion–induced up-regulation of HSP90 and eNOS expression levels was completely abrogated by *HSF1* knockdown in HAEC (Fig. 5C), highlighting the requirement of HSF1 in maintaining the expression of HSP90 and eNOS in ECs with *TXNDC5* knockdown.

Ubiquitin-dependent proteolysis plays an essential role in regulating HSF1 activity and protein levels (*26*). To test whether TXNDC5 regulates HSF1 protein abundance through a ubiquitin/proteasome-dependent mechanism, HSF1 protein was pulled down from control and TXNDC5-overexpressed HAEC lysates and subjected to immunoblotting to assess the extent of HSF1 polyubiquitination. Compared to mock, *TXNDC5* overexpression led to a marked increase in the ubiquitination of HSF1 in HAEC (Fig. 5D and fig. S7D). In addition, *TXNDC5* overexpression–induced HSF1 protein reduction was abolished by the treatment with a proteasome inhibitor MG132 in HAEC (Fig. 5E). These data demonstrate that TXNDC5 regulates HSF1 protein expression by enhancing its polyubiquitination and proteasome-dependent degradation.

In line with the abovementioned in vitro findings, immunoblotting showed a marked up-regulation of t-eNOS, HSP90, and HSF1 proteins in the AA from DKO, compared with *ApoE^−/−^*, mice fed with HFD (Fig. 5F), further demonstrating TXNDC5-dependent suppression of HSF1-HSP90-eNOS in the vasculature in vivo. Together, these data demonstrate that TXNDC5 modulates HSP90 and eNOS expression through the posttranscriptional regulation of HSF1 in the endothelium, where increased TXNDC5 accelerates ubiquitin-dependent HSF1 degradation.

### Endothelial TXNDC5 is transcriptionally repressed by flow-sensitive transcription factor KLF2

**Fig. 6.**
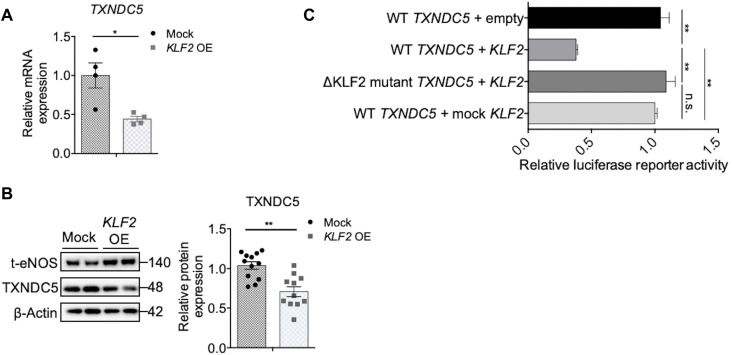
Endothelial TXNDC5 is transcriptionally suppressed by KLF2. Overexpression of *KLF2*, compared to mock (mutant *KLF2* transcripts cannot be translated), led to (**A**) transcriptional repression (*n* = 4) and (**B**) protein down-regulation (*n* = 11 to 12) of TXNDC5 in HAEC. (**C**) WT (*TXNDC5*) and *KLF2* binding site (CACCC; −548 to −544)–deleted (ΔKLF2 mutant *TXNDC5*) human *TXNDC5* promoter luciferase constructs were transfected into HEK293 cells. Overexpression of *KLF2* mRNA significantly reduced WT *TXNDC5* promoter activity, whereas deletion of the KLF2 binding site (ΔKLF2 mutant *TXNDC5*) restored *TXNDC5* promoter activity. In addition, overexpression of mock *KLF2* mRNA (mutant *KLF2* mRNA that cannot be translated) did not affect WT *TXNDC5* promoter activity (*n* = 6) (*P < 0.05 and **P < 0.01 determined using two-tailed Mann-Whitney *U* test).

KLF2, one of the well-characterized flow-sensitive transcription factors, regulates a wide range of atherorelevant genes in the endothelium (*27*–*29*). Because endothelial TXNDC5 is flow sensitive and promoter analysis identified a KLF2 binding motif (CACCC) in both mouse and human *Txndc5/TXNDC5* promoter sequences (fig. S8A), we hypothesized that TXNDC5 could be transcriptionally regulated by KLF2 in the endothelium. Consistent with this hypothesis, overexpression of *KLF2* (fig. S8B), comparing to mock (mutant *KLF2* transcripts that cannot be translated), led to transcriptional repression (Fig. 6A) and protein down-regulation of TXNDC5 (Fig. 6B) in HAEC. Next, WT (*TXNDC5*) and KLF2 binding site (CACCC; −548 to −544)–deleted (ΔKLF2 mutant *TXNDC5*) human *TXNDC5* promoter luciferase constructs were transfected into human embryonic kidney (HEK) 293 cells. As shown in Fig. 6C, overexpression of *KLF2* significantly reduced the transcriptional activity of WT, but not KLF2 binding site–deleted (ΔKLF2 mutant *TXNDC5*) *TXNDC5* promoters. In addition, overexpression of mock *KLF2* (mutant *KLF2* mRNA that cannot be translated) transcript failed to repress *TXNDC5* promoter activity. These data together show that endothelial TXNDC5 is transcriptionally repressed by KLF2 and that DF-induced endothelial TXNDC5 up-regulation is mediated by KLF2 reduction.

### Endothelial *Txndc5* deletion achieved by a targeted nanomedicine platform significantly reduces atherosclerosis in *ApoE^−/−^* mice

**Fig. 7.**
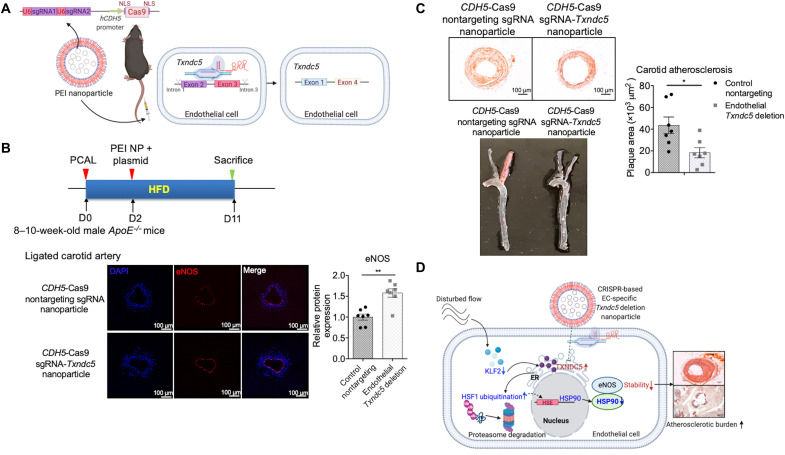
In vivo administration of nanoparticles carrying an endothelium-specific *Txndc5*-targeting CRISPR-Cas9 vector deletes endothelial TXNDC5 and ameliorates DF-induced carotid atherosclerosis. (**A**) Scheme of PEI nanoparticles (NP) carrying a CRISPR-Cas9–based *Txndc5*-targeting vector in which CRISPR-Cas9 expression is driven by the endothelium-specific *CDH5* promoter and sgRNAs targeting *Txndc5* introns 1 and 3 are driven by a U6 promoter. NLS, nuclear localization sequence. (**B**) Single intravenous injection of nanoparticles encapsulating *CDH5*-Cas9/sgRNA-*Txndc5* (2 days after PCAL) significantly restored eNOS protein expression levels (intensity measured along the vessel lumen) in the ligated LCA of *ApoE^−/−^* mice subjected to PCAL/HFD (11 days), compared to those receiving control nanoparticles encapsulating *CDH5*–CRISPR-Cas9 and nontargeting sgRNAs (*n* = 7). (**C**) Treatment with nanoparticles encapsulating *CDH5*-Cas9/sgRNA-*Txndc5* significantly reduced carotid atherosclerosis in *ApoE^−/−^* mice subjected to PCAL/HFD (*n* = 7). (**D**) Schematic summary of the proposed molecular mechanisms by which mechanosensitive TXNDC5 contributes to endothelial activation and atherosclerosis induced by DF, which can be targeted by nanomedicine-mediated genome editing (*P < 0.05 and **P < 0.01 using two-tailed Mann-Whitney *U* test).

To establish a proof of concept to treat atherosclerosis in vivo by reducing DF-induced TXNDC5 in the endothelium, we devised a method that integrates polyethylenimine (PEI) nanoparticles and CRISPR-Cas9–mediated genome editing technique. A plasmid was engineered to express Cas9 driven by a ~2.2-kb human *CDH5* promoter, a sequence shown to drive endothelium-specific expression in vivo (*30*), as well as two U6 promoter-driven single-guide RNAs (sgRNAs) targeting introns 1 and 3 of *Txndc5*, allowing deletion of exon 2/3 (scheme shown in Fig. 7A). Nanoparticles were formulated by complexing cationic polymers PEI and negatively charged plasmids. These nanoparticles were administered in *ApoE^−/−^* mice through a single tail vein injection 2 days (D2) after PCAL (D0), and the animals were euthanized 11 days (D11) after PCAL (time frame of experiments is shown in Fig. 7B). Compared to control nanoparticles encapsulating the plasmid with *CDH5*-Cas9 and nontargeting sgRNAs, *CDH5*-Cas9/sgRNA-*Txndc5*–containing nanoparticles resulted in efficient and specific deletion of endothelial *Txndc5* in the intima, without affecting the *Txndc5* levels in the media + adventitia (M + A), of AA in *ApoE^−/−^* mice (fig. S9). The expression level of eNOS in the ligated LCA was significantly increased in *ApoE^−/−^* mice by endothelial *Txndc5* deletion using *CDH5*-Cas9/sgRNA-*Txndc5* nanoparticles (Fig. 7B). Moreover, DF-induced arterial wall thickening and atherosclerosis in the ligated LCA were significantly reduced in *CDH5*-Cas9/sgRNA-*Txndc5* nanoparticle–treated *ApoE^−/−^* mice, compared to those receiving control nanoparticles (Fig. 7C). These results together demonstrate the effectiveness of this newly devised targeted nanomedicine approach to achieve endothelium-specific *Txndc5* deletion and, moreover, establish the feasibility to spatially manipulate mechanosensitive genes in endothelium treating atherosclerosis in vivo.

## DISCUSSION

Vascular functions and vessel wall homeostasis are dynamically and tightly regulated by mechanotransduction mechanisms, in which ECs sense and convert hemodynamic cues to biological responses (*1*–*3*). Our in vitro and in vivo results collectively demonstrate that mechanosensitive TXNDC5 is a previously unrecognized but key regulator of endothelial activation and atherosclerosis by destabilizing eNOS protein. Moreover, our mechanistic investigations delineate novel molecular insights by which TXNDC5 increases ubiquitination and proteasome-mediated degradation of HSF1, leading to decreased expression of HSP90 required for eNOS protein stability. Capitalizing on these new molecular insights and the in vivo results establishing the causal role of TXNDC5 in atherogenesis, we devised a targeted nanomedicine platform that integrates PEI nanoparticles and CRISPR-Cas9 plasmids with an endothelium-specific promoter (human *CDH5*) (*30*), which specifically deleted *Txndc5* in the endothelium and, moreover, significantly reduced atherosclerosis in *ApoE^−/−^* mice. Collectively, these studies elucidate novel atherorelevant mechanotransduction mechanisms mediated by TXNDC5 in vascular ECs and further establish a proof of concept to target mechanosensitive endothelial TXNDC5 to lessen atherosclerotic diseases in vivo (Fig. 7D).

Although *TXNDC5* was first cloned in human umbilical vein ECs (*13*), the role of endothelial TNXDC5 in mechanotransduction and atherosclerosis has not been reported previously. TXNDC5 is an ER-enriched protein with the enzyme activity of PDI, which facilitates the formation of disulfide bonds and correct folding of nascent polypeptides (*31*). Until this study, the role of endothelial TXNDC5 in vascular disease in vivo remains unexplored, hindered by the lack of any animal model allowing TXNDC5 deletion selectively in the vascular endothelium. Previous in vitro studies demonstrated that TXNDC5 exerts protective effects against hypoxia-induced EC apoptosis and mediates TNFα-induced angiogenesis (*13*, *14*). Supported by complementary in vitro and in vivo results, here we elucidate a new mechanosensitive molecular action of TXNDC5 to destabilize eNOS protein and drive DF-induced atherosclerosis.

Endothelial *NOS3* expression is tightly regulated by hemodynamics and is critical to vascular homeostasis and pathophysiology (*32*–*34*). Our results reveal a new posttranscriptional mechanism by which atheroprotective UF stabilizes, while atheroprone DF destabilizes, eNOS protein. Reduced eNOS expression and consequent NO reduction are key molecular signatures of endothelial dysfunction, leading to a large cohort of vascular diseases including atherosclerosis (*8*, *21*, *22*). Previous studies have shown that eNOS/*NOS3* can be regulated on the transcriptional and posttranscriptional levels in the vascular endothelium (*33*–*36*). UF increases *NOS3* transcription via induction of atheroprotective transcription factors KLF2 and KLF4; UF also stabilizes eNOS protein by enhancing interaction between HSP90 and eNOS (*27*, *28*, *33*). HSP90 was also shown to modulate eNOS protein activity via regulating the conformation of eNOS protein (*37*, *38*), scaffolding eNOS regulatory proteins (*34*), dissociating eNOS from its inhibitors such as caveolin-1 (*24*, *39*), as well as by promoting eNOS dimerization to enhance its enzymatic activity (*40*). However, whether HSP90 expression per se is mechanosensitive and regulated by hemodynamics remained unknown. Our data in this study unveil a new molecular mechanism that TXNDC5 accelerates eNOS protein degradation through transcriptional down-regulation of *HSP90* in response to hemodynamic stress induced by DF.

HSF1 is the major transcriptional regulator of heat shock proteins, and our results provide a new line of evidence demonstrating that HSF1 contributes to the flow-dependent mechanoregulation of endothelium. In addition to thermotolerant response, HSF1 has been implicated in developmental pathways and tissue homeostasis (*41*). HSF1 reduction is also associated with neurodegenerative diseases such as Parkinson’s and Alzheimer’s disease (*26*). Although the mechanosensitivity of endothelial HSF1 has not been suggested previously, several lines of evidence support the atheroprotective role of endothelial HSF1. Statin treatment, for example, induces the phosphorylation and nuclear translocation of HSF1 to promote endothelial health by increasing HSP70, thrombomodulin, and eNOS while suppressing endothelin-1 and plasminogen activator inhibitor-1 (*42*, *43*). Our results demonstrate that TXNDC5 increases HSF1 ubiquitination and proteasome-mediated degradation in ECs under DF. These results are consistent with emerging data, indicating proteasomal degradation as a key molecular control to modulate HSF1 activity (*44*). PDI domain–mutated TXNDC5 loses its capacity to reduce HSF1 protein levels in HAEC, the findings of which were consistent with previous reports showing that chaperone proteins with PDI activity regulate cellular proteostasis through ubiquitin proteasome–dependent mechanisms (*45*–*47*). Nevertheless, the detailed molecular mechanism by which TXNDC5 promotes HSF1 ubiquitination and degradation awaits future studies.

KLF2 is a major transcriptional regulator mediating the anti-inflammatory, vasodilatory, and antithrombotic endothelial phenotype under UF (*27*, *28*, *48*, *49*). Our results demonstrate that TXNDC5 is transcriptionally repressed by KLF2. This result is consistent with previous observations demonstrating that, in addition to transactivation of key anti-inflammatory genes, KLF2 trans-suppresses a cohort of genes critical to endothelial activation (*50*). These data are also in agreement with previous microarray profiling studies showing reduced *TXNDC5* mRNA in KLF2-overexpressed human umbilical cord ECs and increased *Txndc5* transcripts in microvascular endothelium from mice with endothelial deletion of *Klf2/4* (*27*, *51*). Our data suggest a coordinated action of KLF2 to increase *NOS3* activity by direct transcriptional activation of the *NOS3* promoter and by indirect eNOS protein stabilization via TXNDC5 suppression.

Here, we engineered multiple transgenic mouse lines to demonstrate the causal role of endothelial *Txndc5* in eNOS protein reduction and atherosclerosis. Consistent with these findings, TXNDC5 expression is markedly increased in human carotid atherosclerotic lesions, particularly in the intimal region, where eNOS protein expression level is reduced (*22*). Given the importance of flow-dependent regulation of *NOS3* in vascular pathophysiology, we believe that these new mouse lines will be useful in future studies to determine the potential in vivo roles of TXNDC5 in other vascular diseases characterized by reduced eNOS expression, including pulmonary arterial hypertension, aortic aneurysm, and early arteriovenous fistula failure.

Endothelial mechanosensing molecules are attractive targets to mitigate atherogenesis; nevertheless, current atherosclerotic therapies largely treat systemic risk factors but not vasculature per se. Recent discovery of CRISPR-Cas has revolutionized genome editing techniques, shedding light on future cardiovascular gene therapies. For instance, viral delivery of CRISPR-Cas has been used to treat atherosclerosis in mice by targeting lipogenic genes in the liver (*52*, *53*). Leveraging on the efficient CRISPR-Cas–based gene deletion and capitalizing on the critical role of endothelial TXNDC5 in DF-induced atherosclerosis, we devised a new approach integrating nanocarriers and *CDH5*-driven CRISPR-Cas9 plasmids to specifically delete *Txndc5* in vascular endothelium, resulting in increased eNOS protein and reduced atherosclerosis in *ApoE^−/−^* mice. These results not only confirm a major and causal role of endothelial TXNDC5 in eNOS suppression and atherogenesis but also provide a proof of principle using nanoparticle-assisted CRISPR-Cas9 approach to target endothelial mechanosensitive mechanisms treating vascular complications in vivo. Viral and synthetic vectors are candidate carriers to deliver Cas endonuclease (as DNA, RNA, or protein), sgRNA (as synthetic oligonucleotides or expressed plasmids), or the combination of Cas protein and sgRNA (as a single plasmid or ribonucleoprotein complex) (*54*). Synthetic nanoparticles provide several advantages over viral vectors in targeting endothelial mechanotransduction mechanisms in vivo. For example, although AAV is a promising carrier to deliver CRISPR-Cas9 in animal models and clinical trials (*55*), packaging oversized (≥4.8 kb) cargo in AAV is associated with heterogeneous genome fragmentation. In addition, the infectivity of AAV for ECs is relatively low (*56*). Dual AAV system with tissue-specific promoter or tropism modification was used to overcome these barriers; however, the in vivo efficiency of dual AAV system was lower compared to single AAV approaches because of the heterogeneity of cotransduction, especially when administered systemically (*57*, *58*). Exploiting PEI nanoparticles, our approach allows the delivery of CRISPR-Cas9 and two sgRNAs simultaneously in a single plasmid, providing a transient but stable expression of CRISPR-Cas9 compared to nanoparticle-delivered Cas9 ribonucleoproteins and, meanwhile, mitigating possible side effects of genotoxic effects due to the long-term expression of Cas9 using AAV vectors (*59*). Nevertheless, PEI, although well tolerated in mice (*49*, *60*), is not approved by the Food and Drug Administration for human use. Future studies are required to further optimize the formulation of nanoparticles for clinical applications.

In summary, the present study delineates a novel mechanosensing mechanism by which ER protein TXNDC5 promotes endothelial activation and atherogenesis induced by DF. TXNDC5 increases the ubiquitination and proteasome-mediated degradation of transcription factor HSF1, leading to decreased HSP90 expression and reduced eNOS protein stability. Endothelial TXNDC5-dependent eNOS protein regulation and atherosclerosis in vivo were demonstrated using new transgenic mouse lines and supported by increased TXNDC5 expression detected in human atherosclerotic lesions. Nanoparticle-delivered, CRISPR-Cas9–mediated endothelial *Txndc5* deletion significantly reduced atherosclerosis in mice. These results collectively demonstrate that targeting mechanosensitive endothelial TXNDC5 could be a novel therapeutic approach to treat or prevent atherosclerotic cardiovascular diseases.

## MATERIALS AND METHODS

All studies were conducted in accordance with protocols approved by the Institutional Review Boards of National Taiwan University and the University of Chicago. All experimental animals were assigned unique identifiers to blind experimenter to genotypes and treatment. A block randomization method was used to assign experimental animals to groups on a rolling basis to achieve adequate sample number for each experimental condition.

### Generation of *Txndc5^−/−^*, *Txndc5^fl/fl^* mice, and cell type–specific *Txndc5* conditional knockout mice

The generation of *Txndc5^−/−^* and *Txndc5^fl/fl^* mice has been described previously (*15*, *16*). To generate an inducible, endothelial-specific, conditional *Txndc5* mouse line, *Txndc5^fl/fl^* mice were bred with *Cdh5-Cre/ERT2* (provided by R. H. Adams, London, UK; to generate *Txndc5*^∆ECKO^) transgenic mice. For activation of the Cre-ERT system, tamoxifen (2 mg/day; dissolved in corn oil or olive oil) was injected intraperitoneally (ip) for five consecutive days at the age of 5 to 6 weeks in control (*Cdh5-Cre/ERT2*) and *Txndc5*^∆ECKO^ mice. The induction of Cre recombinase for efficient deletion of *Txndc5* was described in Results.

### In vivo deletion of endothelial *Txndc5* using CRISPR-Cas9

*ApoE^−/−^* mice, 8 to 10 weeks old, were subjected to PCAL (D0), followed by tail vein injection with the mixture of PEI nanoparticles (*49*, *60*) (TurboFect, Thermo Fisher Scientific, MA, USA) and plasmid 2 days later (D2). The mixture consisted of 40 μg of endotoxin-free plasmid DNA containing either a CRISPR-Cas9–based *Txndc5*-targeting vector (*CDH5*-Cas9/sgRNA-*Txndc5*), where sgRNAs targeting introns 1 and 3 of *Txndc5* that allowed deletion of exon 2/3 were driven by the U6 promoter in the same vector (scheme shown in Fig. 7A), or a CRISPR-Cas9–based nontargeting vector, sterile 5% glucose water, and TurboFect in vivo transfection reagent (Thermo Fisher Scientific, MA, USA). After PCAL, the *ApoE^−/−^* mice were fed with HFD for a total of 11 days, and carotid arteries and blood were collected for downstream experiments.

### Reporter mouse model

The EC-specific tdTomato-expressing mouse model (*Tie2-tdTomato*) was generated by crossing *Tie2-Cre/ERT2* with *ROSA26-tdTomato* mice in *ApoE^−/−^* background. In these animals, ECs were labeled with tdTomato after tamoxifen induction (80 mg/kg/day, ip, for 5 days).

### PCAL surgery

To perform PCAL, anesthesia was first induced by intraperitoneal injection of 2,2,2-tribromeethanol (250 mg/kg, ip) or mixture of ketamine (100 mg/kg, ip) and xylazine (10 mg/kg, ip). After shaving, a ventral midline incision (4 to 5 mm) was made in the neck. LCA was exposed by blunt dissection. Three of four caudal branches of LCA—left external carotid artery, left internal carotid artery, and occipital artery—were ligated with a 6-0 silk suture, while the superior thyroid artery was left intact (scheme shown in Fig. 1B). The incision was then closed, and mice were monitored in a chamber on a heating pad following surgery until full recovery. For *ApoE^−/−^* and DKO mice, mice were fed with HFD [21% fat and 0.2% cholesterol (% by weight); Harlan-Envigo, IN, USA, TD.88137] following PCAL, and both LCA and right carotid artery (RCA) were harvested 2 weeks after PCAL (*18*). For *Cdh5-Cre/ERT2* and *Txndc5*^∆ECKO^, mice were subjected to PCAL 1 week after AAV9-PCSK9 injection and fed with HFD for another 3 weeks (scheme shown in Fig. 2E). At end points, both LCA and RCA were harvested for downstream experiments.

### Intimal RNA isolation from carotid arteries

After careful isolation, the carotid lumen was quickly flushed with 350 μl of QIAzol lysis reagent (QIAGEN, MD, USA) using a 29-gauge insulin syringe, and the elute was collected in a microfuge tube. The elute was further applied for intimal RNA isolation. The carotid artery leftover after flushing with QIAzol was homogenized with 700 μl of QIAzol for RNA isolation of media and adventitia. Reverse transcription and qRT-PCR were performed using methods described previously (*15*). Mouse PCR primers are listed in table S2.

### AAV9-PCSK9 mouse model

Three weeks after tamoxifen induction, recombinant AAV serotype-9 expressing the PCSK9 mutant under the hepatic control region-apolipoprotein enhancer/alpha1-antitrypsin, a liver-specific promoter [AAV9-HCRApoE/hAAT-D377Y-mPCSK9; 1 × 10^11^ viral genomes (VG)] was injected via tail vein and fed with HFD both in *Txndc5*^∆ECKO^ and *Cdh5-Cre/ERT2*. One week after AAV9-PCSK9 injection, mice were subjected to PCAL, and HFD was continued for another 3 weeks, an established model of accelerated carotid atherosclerosis within 3 to 4 weeks (scheme shown in Fig. 2E) (*20*). Then, the animals were euthanized, blood was collected, and carotid arteries and heart were harvested after saline perfusion via the left ventricle after severing the inferior vena cava.

### Plaque lesion analysis of aorta, aortic sinuses, and carotid arteries

After euthanasia and saline perfusion, aorta, heart, and carotid arteries were isolated en bloc. Heart and carotid arteries were fixed in 4% paraformaldehyde, sequentially immersed in 30% sucrose and optimal cutting temperature compound (OCT):30% sucrose (1:1) mixed solution, and then embedded in OCT. Frozen-embedded samples were sectioned in 5- to 8-μm thickness and stained with an Oil Red O staining kit (Sigma-Aldrich, MO, USA and ScienCell, CA, USA) according to the manufacturers’ instructions. The aorta was fixed in 4% paraformaldehyde for 16 hours and then stained with an Oil Red O staining kit. The plaque areas were calculated using the National Institutes of Health ImageJ software. The extent of atherosclerosis in the en face aorta was presented as the plaque areas relative to the total aortic area, while those in the carotid arteries and aortic sinuses were presented as the absolute plaque areas on each frozen section.

### IF staining

Frozen samples were sectioned in 5 to 8 μm, permeabilized, and blocked with 1% bovine serum albumin (BSA) for 1 hour. Fixed samples were then incubated with primary antibodies (described in table S3) overnight at 4°C. After washing, the sectioned samples were incubated with Alexa Fluor 594–labeled anti-rabbit and/or Alexa Fluor 488–labeled anti-mouse secondary antibodies (1:250 to 1:500; BioLegend, CA, USA) at room temperature for 1 hour. The sections were then washed and mounted with ProLong Gold (Thermo Fisher Scientific, MA, USA). Fluorescence imaging was acquired using a Zeiss Axio Imager M1 fluorescence microscope (Carl Zeiss, Oberkochen, Germany) and analyzed using iVision image analysis software (BioVision Technologies, PA, USA) and ImageJ.

### En face staining

Aortas of C57BL/6 mice were fixed with 4% paraformaldehyde through systemic perfusion for 5 min. After permeabilization/blocking in 0.1% Triton X-100 [in phosphate-buffered saline (PBS)] for 10 min and 10% BSA for 30 min at room temperature, aortas were incubated at 4°C overnight in incubation buffer containing 10% BSA and the primary antibodies (described in table S3). After washing with PBS for three times, aortas were incubated with Alexa Fluor 594–labeled anti-rabbit and/or Alexa Fluor 488–labeled anti-mouse secondary antibodies (1:500 to 1:1000; BioLegend, CA, USA) at room temperature for 1 hour. Fluorescence imaging was acquired using a Zeiss Axio Imager M1 fluorescence microscope (Carl Zeiss, Oberkochen, Germany) and analyzed using iVision image analysis software (BioVision Technologies, PA, USA) and ImageJ.

### HAEC culture

Primary HAEC were purchased from Lonza (NJ, USA) (CC-2535, lot number 0000633456) and cultured in EGM-2 supplemented with SingleQuots (Lonza, CC-3156 and CC-4176) and antibiotic-antimycotic (Gibco, NY, US) in a humidified atmosphere of room air supplemented with 95% O_2_/5% CO_2_ at 37°C.

### Atherorelevant cone plate flow system

Cells were seeded in six-well plates at the density of 4 × 10^5^ cells per well. HAEC at 100% confluence, maintained in EGM-2 medium containing 4% dextran (Sigma-Aldrich, MO, USA) in six-well plates, were subjected to atheroprotective or atherosusceptible flow waveform for 24 hours under normal culture condition (*11*).

### siRNA transfection of HAEC

Control siRNAs (siControl; QIAGEN, MD, USA, #1027310) and siRNAs targeting *TXNDC5* (si*TXNDC5*; QIAGEN, MD, USA, #SI00132447) were used for *TXNDC5* knockdown. Transfection was performed using 50 nM siRNA with Lipofectamine RNAiMAX (Thermo Fisher Scientific, MA, USA) according to the manufacturer’s instructions. Knockdown efficiency was assayed using qRT-PCR.

### Lentiviral transduction of HAEC

HAEC were transduced using lentiviral vectors carrying a puromycin-resistant gene (15 multiplicity of infection) for 24 hours, and the transduced cells were selected using puromycin (1 ng/ml) for 48 hours. Transduction efficiency was assayed using qRT-PCR.

For knockdown experiment, lentiviral particles containing short hairpin RNAs (shRNAs) pLKO-sh*HSP90aa1* (#TRCN0000315009), pLKO-sh*TXNDC5* (#TRCN000033258), and pLKO-sh*HSF1* (#TRCN0000007484) were used for knockdown of *HSP90aa1* (sh*HSP90aa1*), *TXNDC5* (sh*TXNDC5*), and *HSF1* (sh*HSF1*) in HAEC, respectively. Lentiviral particles containing scrambled shRNA, pLKO-shScr (#TRCN00001), were used as nontargeting control (shScr). For overexpression experiment, lentiviral particles containing human *TXNDC5* gene (pLAS2w.pPuro-*TXNDC5*) and empty pLAS2w.pPuro vector were used for overexpression of *TXNDC5* and control in HAEC, respectively.

### mRNA transfection of HAEC

In vitro transcription for WT (T7-*KLF2*), ATG-mutated *KLF2* (T7-mock), *TXNDC5*, and cysteine mutant *TXNDC5* mRNA transcripts was performed using the mMESSAGE mMACHINE T7 Ultra Kit (Thermo Fisher Scientific, MA, USA). HAEC were transfected with *KLF2*, *KLF2*-mock, *TXNDC5*, and cysteine-mutated *TXNDC5* mRNA transcripts using the Lipofectamine MessengerMAX (Thermo Fisher Scientific, MA, USA) according to the manufacturer’s instructions.

### RNA extraction and qRT-PCR

Total RNA was isolated from HAEC using TRIzol (Thermo Fisher Scientific, MA, USA) according to the manufacturer’s recommendations. Total RNA was reverse transcribed with the Maxima First Strand cDNA Synthesis Kit (Thermo Fisher Scientific, MA, USA), and qRT-PCR was performed using SYBR Green (Bio-Rad Laboratories, CA, USA). Human and mouse PCR primers are listed in table S2. The expression levels of individual transcripts were normalized to control gene hypoxanthine-guanine phosphoribosyltransferase and expressed relative to the mean expression values of control samples.

### Immunoblot analysis

Aortic tissues and cells were lysed either in 1× Cell Lysis Buffer (Cell Signaling Technology, MA, USA) supplemented with protease inhibitor cocktail and HALT phosphatase inhibitors (Thermo Fisher Scientific, MA, USA) or in urea buffer containing 8 M deionized urea, 1% SDS, 10% glycerol, 60 mM tris-HCl, and 5% 2-mercaptoethanol (all chemicals were from Sigma-Aldrich, MO, USA). The lysates from the cell lysis buffer were then centrifuged at 4°C for 10 min at 10,000*g*, and the supernatant was mixed with 4× protein loading buffer. Protein lysates were boiled at 95°C for 5 min, and the concentration of protein lysate was determined using BCA protein assay. The lysates from urea buffer were passed through a 28-gauge insulin syringe five times and then centrifuged at 4°C for 10 min at 10,000*g*. Aortic tissue homogenates and cell lysates were fractionated on 10% SDS–polyacrylamide gel electrophoresis gel, transferred onto a polyvinylidene difluoride membrane, and then blocked in blocking buffer (5% BSA and 0.1% Tween 20 in PBS). Membranes were incubated with primary antibodies, as described in table S3, overnight at 4°C. Blots were developed using horseradish peroxidase (HRP)–conjugated anti-mouse or anti-rabbit whole immunoglobulin G secondary antibodies (1:10,000; Thermo Fisher Scientific, MA, USA) and WesternBright ECL HRP substrate (Advansta, CA, USA). Protein band detection was performed using the ChemiDoc MP system (Bio-Rad Laboratories, CA, USA). Protein band intensity quantification analysis was performed with ImageLab software version 5.1.

### Cycloheximide protein stability assay

siControl and si*TXNDC5*-transfected HAEC were treated with cycloheximide (25 μg/ml) (Sigma Aldrich, MO, USA) to inhibit protein translation. The eNOS protein levels were determined in both siControl- and si*TXNDC5*-transfected HAEC at 0, 4, and 10 hours after cycloheximide treatment.

### Coimmunoprecipitation for TXNDC5-interacting proteins and ubiquitination of HSF1

Immunoprecipitation (IP) of target proteins was conducted using a magnetic IP kit (Thermo Fisher Scientific, MA, USA, #88804). Protein lysates from HAEC with a total of 1000 μg of protein were incubated with 10 μg of primary antibodies against TXNDC5 (Proteintech, IL, USA, #19834-1-AP) or HSF1 (Cell Signaling Technology, MA, USA, #4356) overnight at 4°C. The immune complex was bound to protein A/G magnetic beads and collected with a magnetic stand. Proteins coimmunoprecipitated with the target protein prepared the day before were eluted and subjected to gel electrophoresis and immunoblotting using antibodies against HSF1 or ubiquitin (1:1000; Cell Signaling Technology, MA, USA, #3936).

### TXNDC5 promoter luciferase reporter assay

*TXNDC5* promoter luciferase activity assay was implemented according to the Luc-Pair Duo-Luciferase Assay Kit 2.0 user manual (GeneCopoeia, MD, USA). HAEC were transfected with luciferase reporter constructs containing *TXNDC5* promotor with (pGL3-hTXNDC5, −548 to −544 deleted, ΔKLF2 mutant *TXNDC5*) or without (pGL3-hTXNDC5, −1000 to +1000) deletion of its *KLF2* binding site, and WT or ATG-mutated *KLF2* (mock *KLF2*) *KLF2* mRNA for 24 hours. After cell lysis and incubation with Luc I and Luc II buffers, the signal intensity of luciferase activity was evaluated in a plate reader (Synergy HT, BioTek, VT, USA). In these experiments, a *Renilla* luciferase expression vector was cotransfected and used as a control for transfection efficiency.

### NO production assay

A nitrate/nitrite fluorometric assay kit (Cayman Chemical, MI, USA) was used to measure the NO production of HAEC. shScr or sh*TXNDC5*, as well as empty vector– or *TXNDC5* vector–transduced HAECs were seeded into a six-well plate at the density of 10^5^ cells per well, and then cultured for 24 hours. A total of 20 μl of the culture medium from each well was collected for NO measurement following the manufacturer’s instructions.

### Transendothelial electrical resistance

TEER was measured using the Millicell ERS-2 Volt-Ohm Meter (Merck, MA, USA) following the manufacturer’s instructions. Endothelial impedance was presented as unit area resistance (ohm·cm^2^).

### RNA-seq and pathway enrichment analysis

Sequence read pairs were aligned to the human genome (hg38) with HISAT2 (*61*), followed by processing and sorting with SAMtools (*62*). The processed files were then imported into Partek Flow (Partek, MO, USA) to quantify gene expression using the RefSeq annotation database. The raw fragment counts of individual mRNA were normalized to the transcript length (in kilobase) and total mapped fragment counts (in million fragments) in the same sample and expressed as FPKM (fragments per kilobase per million mapped fragments). Differential expression analyses were performed using DESeq2 (*63*), and the cutoff for significance was set at a false discovery rate–adjusted *P* < 0.05.

GO and pathway enrichment analyses were performed using WebGestalt (Web-based Gene Set Analysis Toolkit; www.webgestalt.org), a web-based software for gene set functional enrichment analysis. The 71 genes that were up-regulated significantly in *TXNDC5*-depleted (si*TXNDC5*), compared to siScr-treated, HAEC exposed to DF were uploaded to WebGestalt, where GO and pathway (Kyoto Encyclopedia of Genes and Genomes and Reactome databases) enrichment analyses were performed using the reference gene set from affy hg u133 plus 2. The fold enrichment and significance level of the enriched GO terms and pathway were plotted and presented in fig. S5 (B and C).

### Reanalysis of publicly available microarray datasets

Microarray datasets GSE100927 (human arterial tissues), GSE28829 (human arterial plaques), and GSE69187 (aorta from HFD-fed or CD-fed *Ldlr^−/−^* mice) are publicly available in the National Center for Biotechnology Information Gene Expression Omnibus (GEO) database (www.ncbi.nlm.nih.gov/geo). The built-in GEO2R function on the GEO website was applied to extract the transcript expression values of TXNDC5 (in a logarithmic scale) from each dataset. After linear transformation, the transcript expression values of TXNDC5 were plotted and compared between groups using GraphPad Prism 6.0.

### Statistical analysis

All experimental data were reported as means ± SEM. The statistical significance of differences between experimental groups was evaluated by Mann-Whitney *U* test. *P* values <0.05 were considered statistically significant.

## Notes

View/request a protocol for this paper from *Bio-protocol*.
