# Supplementary material for: Targeting mechanosensitive endothelial TXNDC5 to stabilize eNOS and reduce atherosclerosis in vivo
Source: Sci Adv. 2022 Jan 21;8(3):eabl8096. doi: 10.1126/sciadv.abl8096 (PMC8782452; doi:10.1126/sciadv.abl8096)
Supplement: Supplementary file 1 — Figs. S1 to S9 Tables S1 to S3 [file sciadv.abl8096_sm.pdf]

Science Advances
AAAS

Supplementary Materials for

Targeting mechanosensitive endothelial TXNDC5 to stabilize eNOS and reduce atherosclerosis in vivo

Chih-Fan Yeh, Shih-Hsin Cheng, Yu-Shan Lin, Tzu-Pin Shentu, Ru-Ting Huang, Jiayu Zhu, Yen-Ting Chen, Sandeep Kumar, Mao-Shin Lin, Hsien-Li Kao, Po-Hsun Huang, Esther Roselló-Sastre, Francisca Garcia, Hanjoong Jo, Yun Fang*, Kai-Chien Yang*

*Corresponding author. Email: kcyang@ntu.edu.tw (K.-C.Y.); yfang1@medicine.bsd.uchicago.edu (Y.F.)

Published 21 January 2022, *Sci. Adv.* **8**, eabl8096 (2022)
DOI: 10.1126/sciadv.abl8096

This PDF file includes:

Figs. S1 to S9
Tables S1 to S3

Figure S1

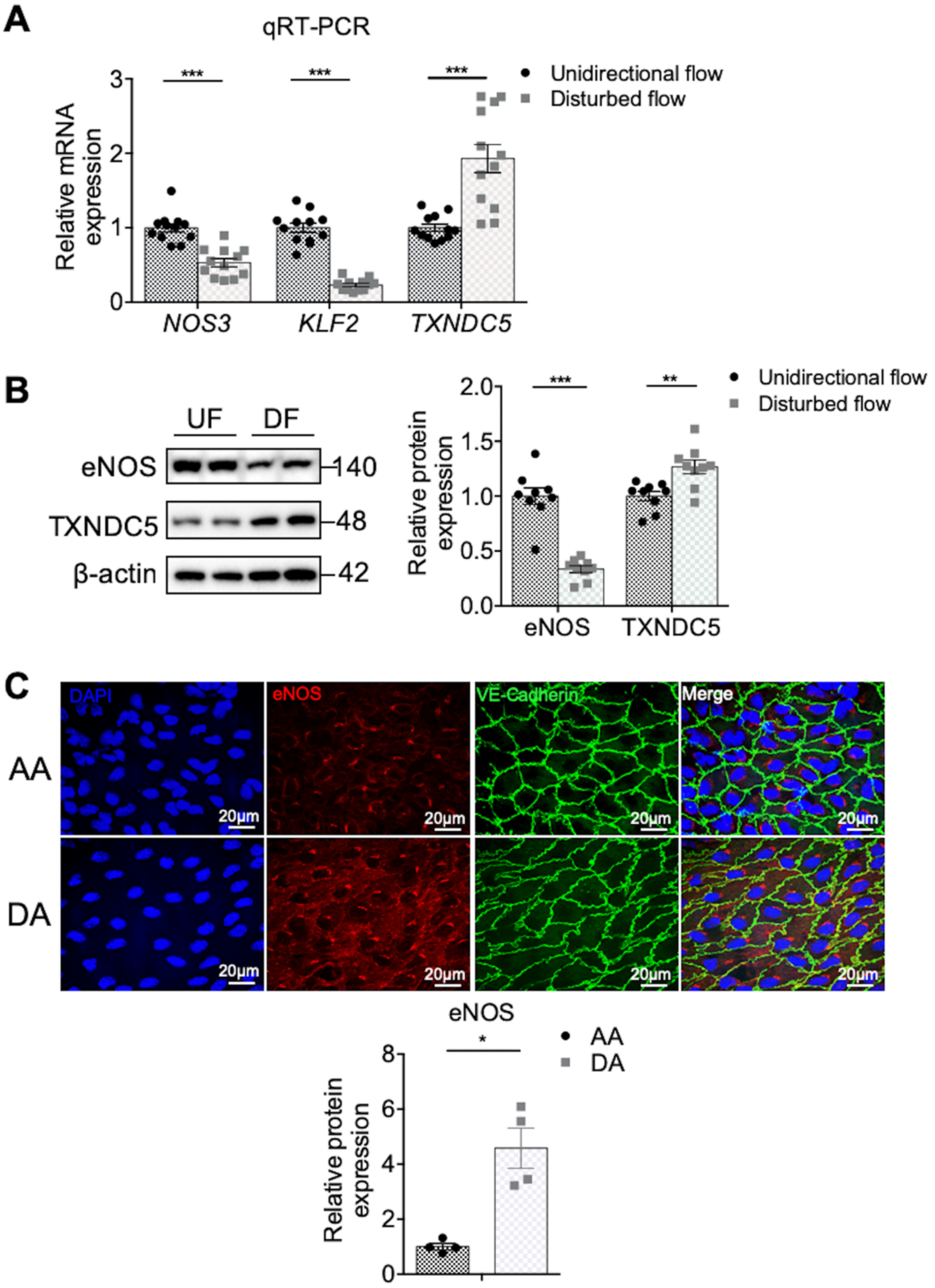

Figure S1. TXNDC5 expression is significantly upregulated in endothelial cells exposed to disturbed flow. (A) qRT-PCR (n=12) and **(B)** immunoblotting (n=9) showed upregulation of TXNDC5 transcript and protein levels, accompanied by downregulation of *NOS3*/eNOS and *KLF2*, in HAEC subjected to 24-hr athero-susceptible disturbed flow (DF) when compared to those in cells under 24-hr athero-protective unidirectional flow (UF). **(C)** *En face* staining of the mouse aorta showed decreased eNOS expression in the endothelium of aortic arch (AA, inner curvature) compared to that of descending thoracic aorta (DA) in C57BL/6 mice (n=4). (* denotes p<0.05, ** denotes p<0.01, *** denotes p<0.001 determined using two-tailed Mann-

Whitney U test).

Figure S2

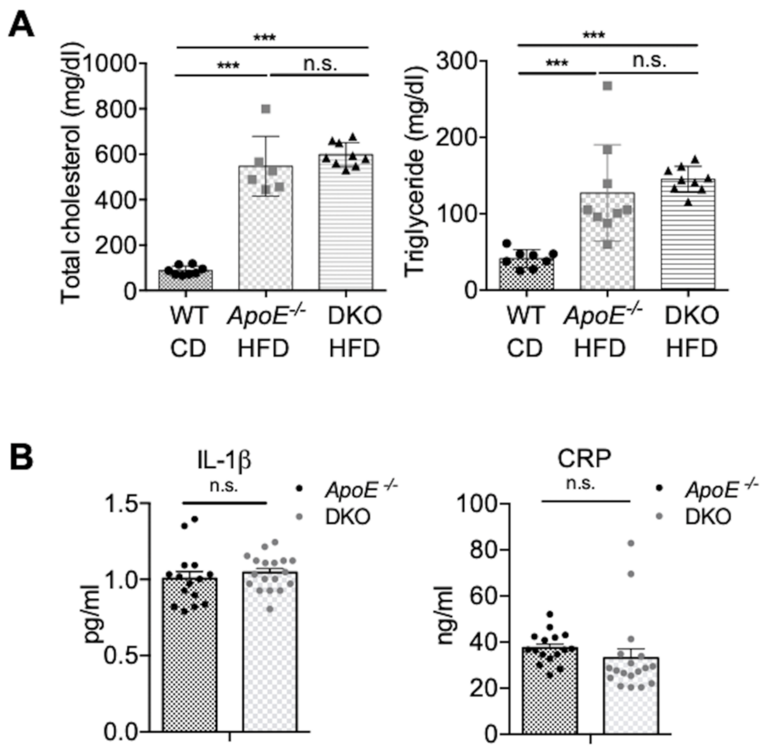

Figure S2. No change of plasma lipid profile or inflammatory markers by *Txndc5* deletion in *ApoE$^{-/-}$* mice. (A) Plasma levels of total cholesterol and triglyceride were similar in DKO and *ApoE$^{-/-}$* mice fed with HFD (n=6-9). Wild type (WT): C57BL/6 mice **(B)** Plasma levels of IL1β and C-reactive protein (CRP) were indistinguishable between *ApoE$^{-/-}$* and DKO mice fed with HFD (n=15-18). (*** denotes p<0.001, n.s.=non-significant determined using two-tailed Mann-Whitney U test).

Figure S3

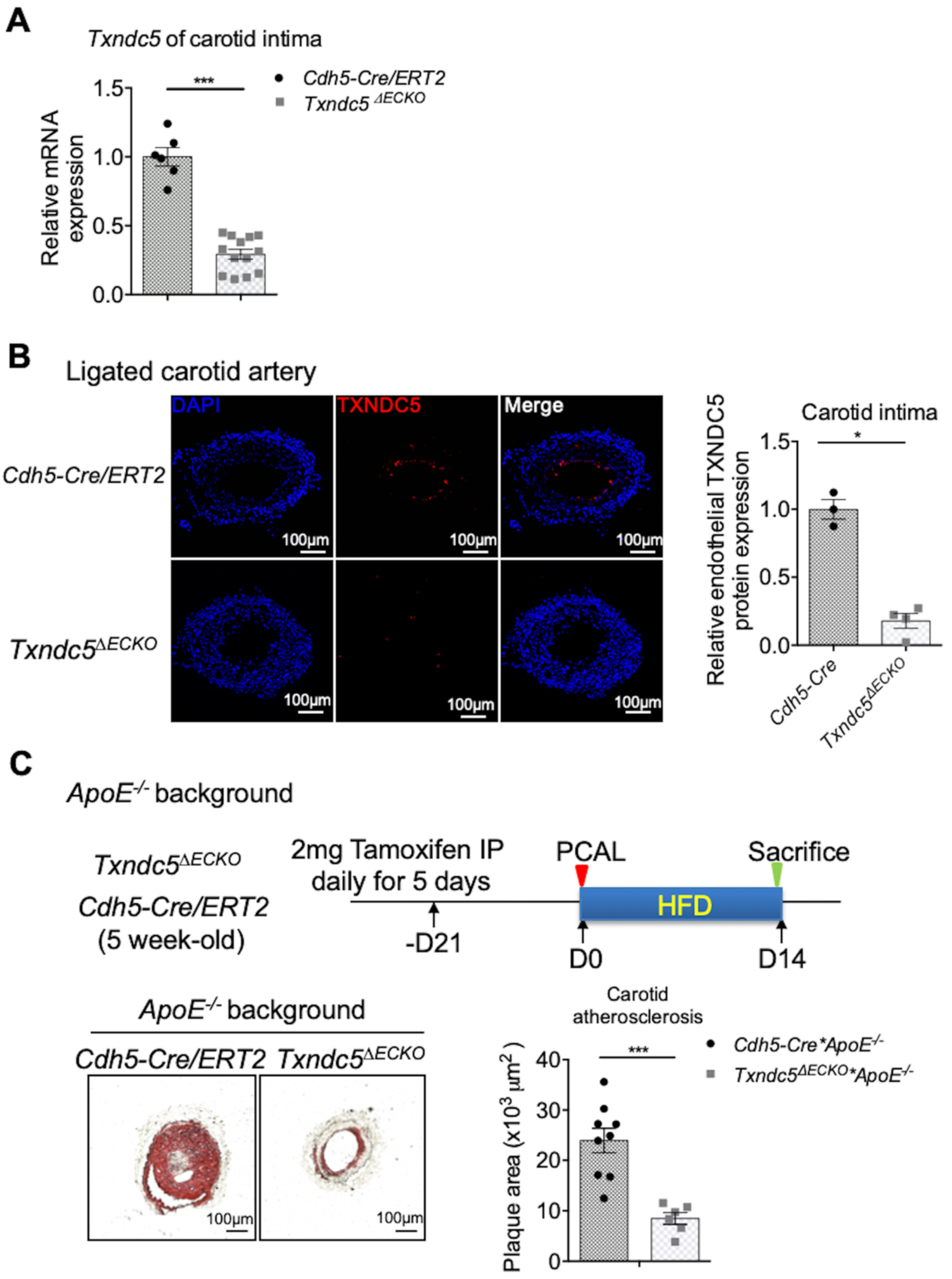

A
Txndc5 of carotid intima
Relative mRNA expression
***
Cdh5-Cre/ERT2
Txndc5 ΔECKO
1.5
1.0
0.5
0.0
B
Ligated carotid artery
DAPI
TXNDC5
Merge
Cdh5-Cre/ERT2
Txndc5ΔECKO
100µm
Carotid intima
Relative endothelial TXNDC5 protein expression
*
Cdh5-Cre
Txndc5ΔECKO
C
ApoE-/- background
Txndc5ΔECKO
Cdh5-Cre/ERT2
(5 week-old)
2mg Tamoxifen IP daily for 5 days
PCAL
Sacrifice
HFD
-D21
D0
D14
ApoE-/- background
Cdh5-Cre/ERT2
Txndc5ΔECKO
100µm
Carotid atherosclerosis
Plaque area (x10³ µm²)
***
Cdh5-Cre*ApoE-/-
Txndc5ΔECKO*ApoE-/-
40
30
20
10
0

Figure S3 (continued)

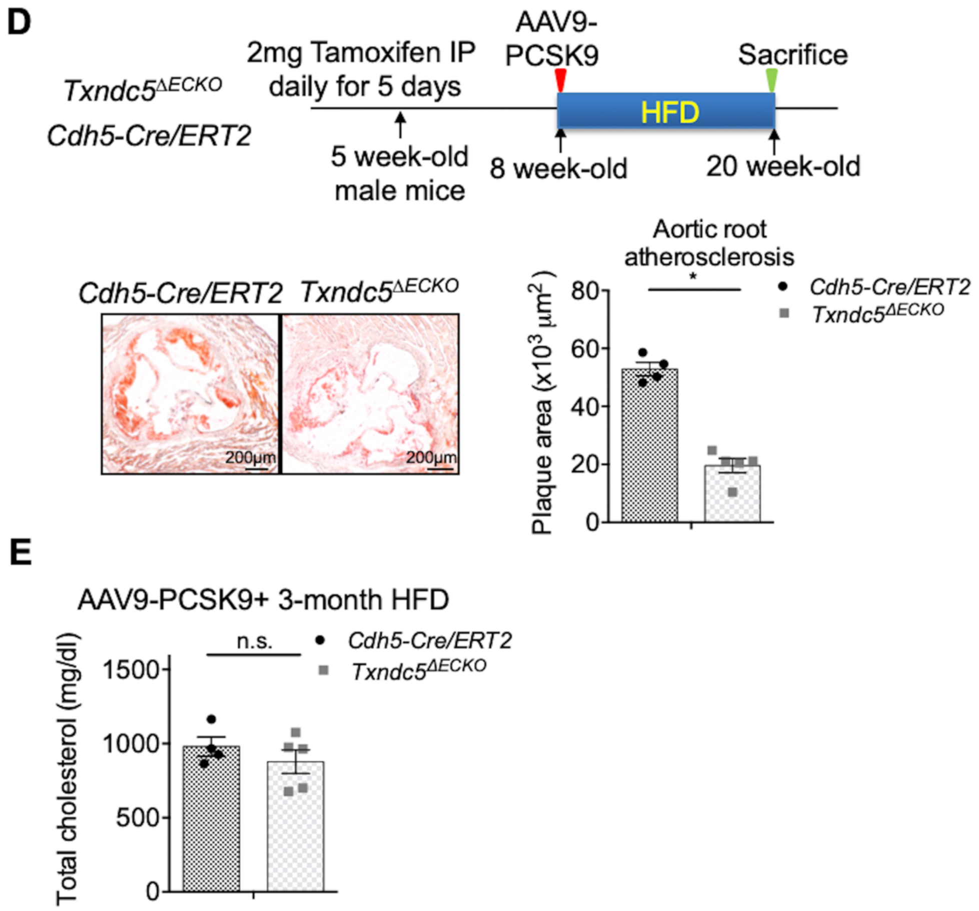

Figure S3. Endothelial deletion of disturbed flow-induced *Txndc5* significantly reduces atherosclerosis *in vivo*. (A-B) *Txndc5* was efficiently deleted in the endothelium-enriched intima of contralateral (RNA expression) (n=6-13) and ligated carotid arteries (protein expression using IF staining, intensity measured along the vessel lumen, n=3-4) from *Cdh5-Cre/ERT2::*$Txndc5^{fl/fl}$ ($Txndc5^{\Delta ECKO}$) mice upon tamoxifen injection. **(C)** Endothelial-specific deletion of *Txndc5* by *Cdh5-Cre* recombinase significantly reduced DF-induced atherosclerosis in the ligated LCA in hypercholesterolemic *Cdh5-Cre/ERT2::*$Txndc5^{fl/fl}$ ($Txndc5^{\Delta ECKO}$) mice compared to tamoxifen-treated *Cdh5-Cre/ERT2* controls in the $ApoE^{-/-}$ background (n=6-9). **(D)** Endothelium-specific deletion of *Txndc5* significantly reduced atherosclerotic lesions at aortic sinus in hypercholesterolemic $Txndc5^{\Delta ECKO}$ mice compared to tamoxifen-treated *Cdh5-Cre/ERT2* controls. Hypercholesterolemia was induced by *PCSK9*-overexpression (one tail vein injection of AAV9-PCSK9, 1×10^{11} VG) and 3-month HFD (n=4-5). **(E)** No significant differences in total plasma cholesterol levels were observed in $Txndc5^{\Delta ECKO}$ and *Cdh5-Cre/ERT2* mice subjected to AAV9-PCSK9 injection and fed with 3-month HFD (n=4-5) (* denotes p<0.05, *** denotes p<0.001, n.s.=non-significant determined using two-tailed Mann-Whitney U test).

Figure S4

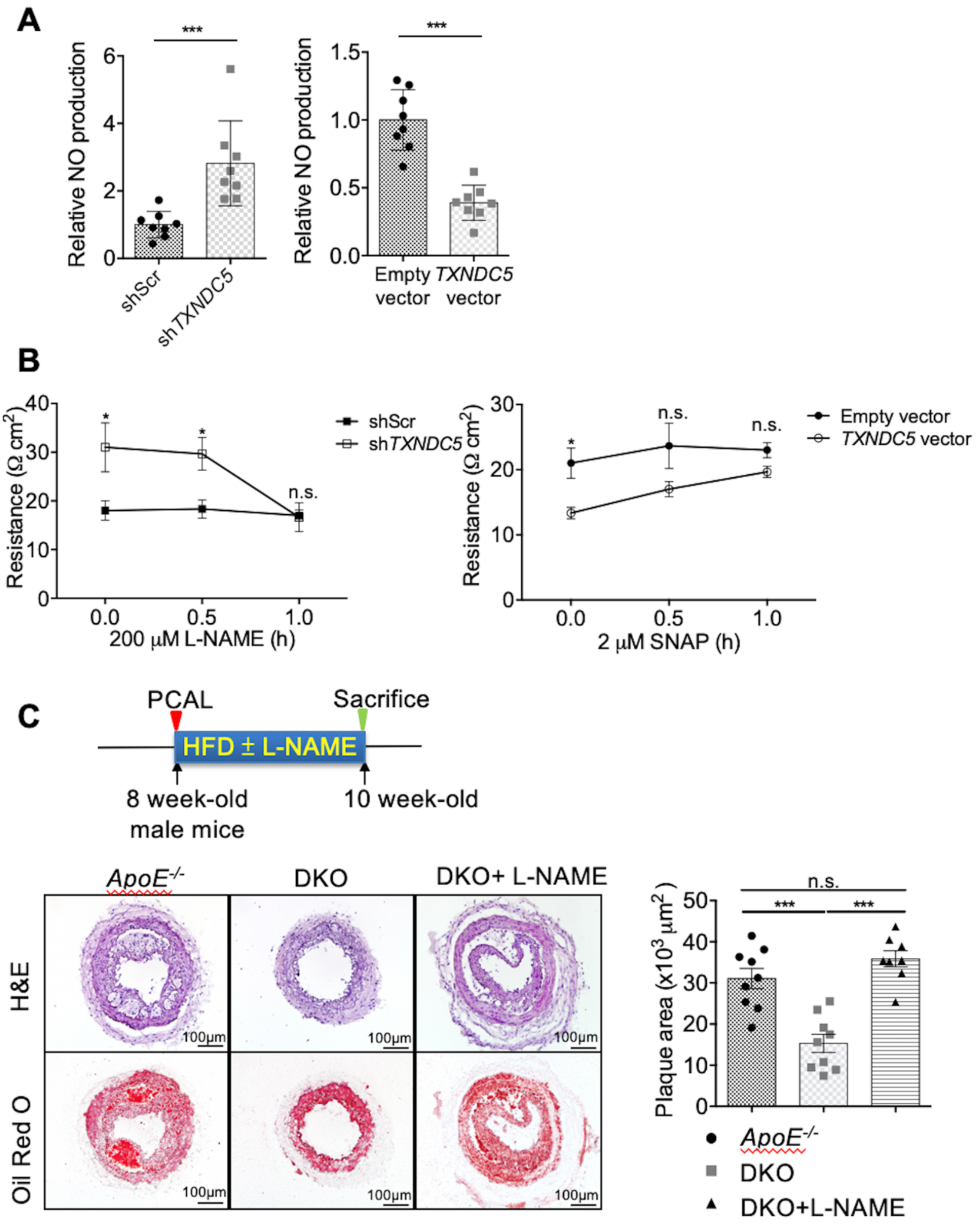

Figure S4. TXNDC5-mediated endothelial dysfunction and atherosclerosis are eNOS/nitric oxide-dependent. (A) Nitric oxide (NO) production was significantly increased in *TXNDC5*-knockdowned (with shRNA, sh*TXNDC5*), whereas decreased in *TXNDC5*-overexpressed (*TXNDC5* vector), HAEC (n=8). **(B)** Knockdown of *TXNDC5* increased, while overexpression of *TXNDC5* reduced, transendothelial electrical resistance (TEER) measured in HAEC. *TXNDC5* knockdown-induced increase in TEER was blocked by the treatment of NOS inhibitor L-N^G-nitroarginine methyl ester

(L-NAME, 200 μM), whereas *TXNDC5* overexpression-mediated TEER reduction was mitigated by the treatment with an NO donor S-Nitroso-N-Acetyl-D, L-Penicillamine (SNAP, 2μM) in HAEC (n=3). **(C)** Global *Txndc5* deletion significantly reduced DF-induced atherosclerosis in the ligated carotid artery in DKO (*Txndc5*$^{-/-}$::*ApoE*$^{-/-}$) mice compared to *ApoE*$^{-/-}$ mice. Inhibition of eNOS activity *in vivo* using L-NAME (4.3mmol/L in drinking water) simultaneously with HFD abrogated the reduction of atherosclerotic plaques caused by *Txndc5* deletion in *ApoE*$^{-/-}$ mice following 2-week PCAL+HFD (n=8-9). (*denotes p<0.05, ***denotes p<0.001, n.s.=non-significant determined using two-tailed Mann-Whitney U test in Figure A and C, and unpaired t-test in Figure B).

Figure S5

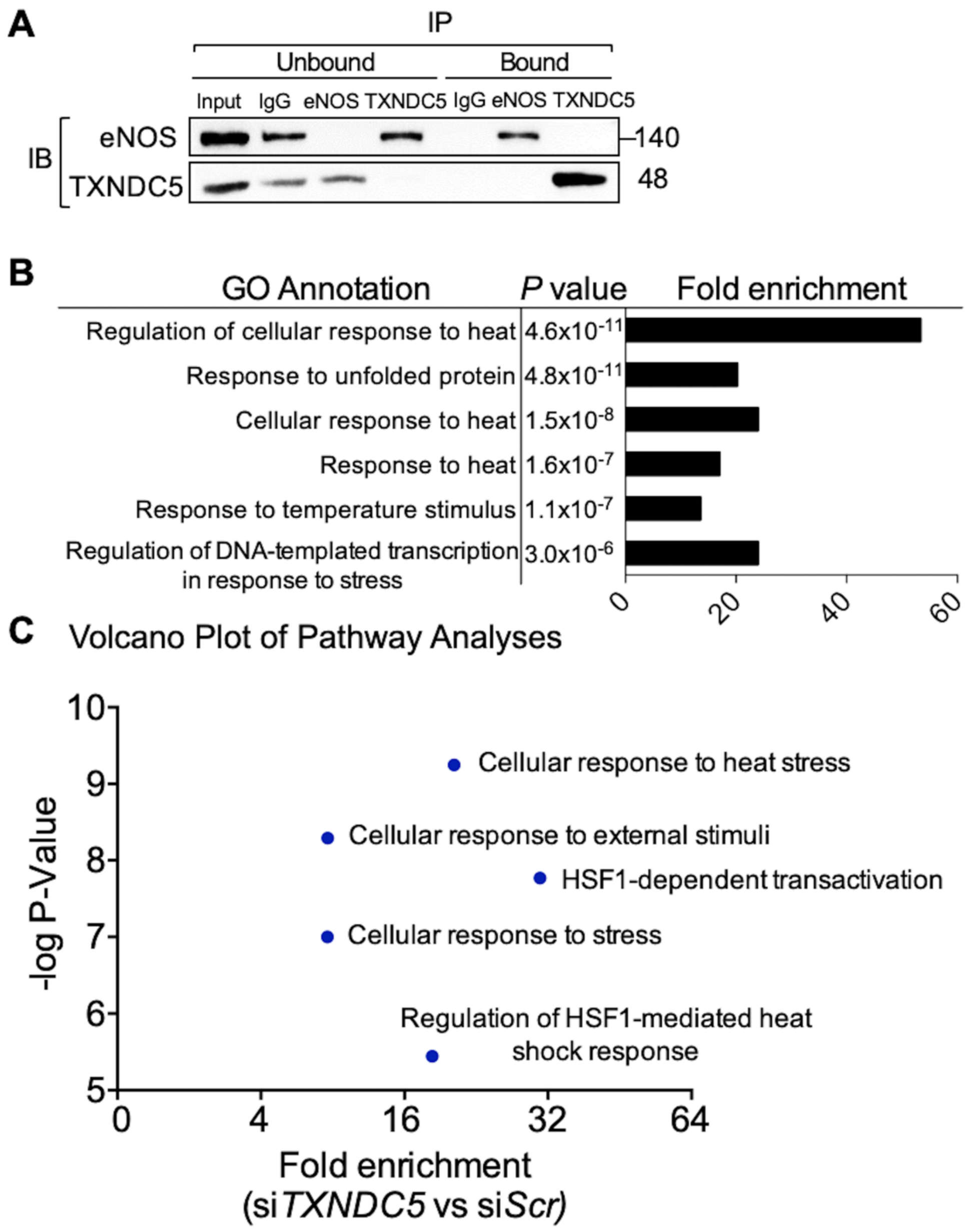

Figure S5. RNA sequencing analysis identifies heat shock response regulated by TXNDC5 depletion. **(A)** Co-immunoprecipitation (Co-IP) experiments showed no physical interaction between TXNDC5 and eNOS. **(B)** Gene ontology (GO) and **(C)** pathway analyses of the transcripts that were upregulated in *TXNDC5*-depleted (si*TXNDC5*) HAEC exposed to DF revealed a significant enrichment of genes that are involved in heat shock response, including regulation of HSF1 (heat shock factor 1)-mediated heat shock responses, cellular response to heat/external stimuli, and response to unfolded protein. siScr: non-targeting scrambled control.

Figure S6

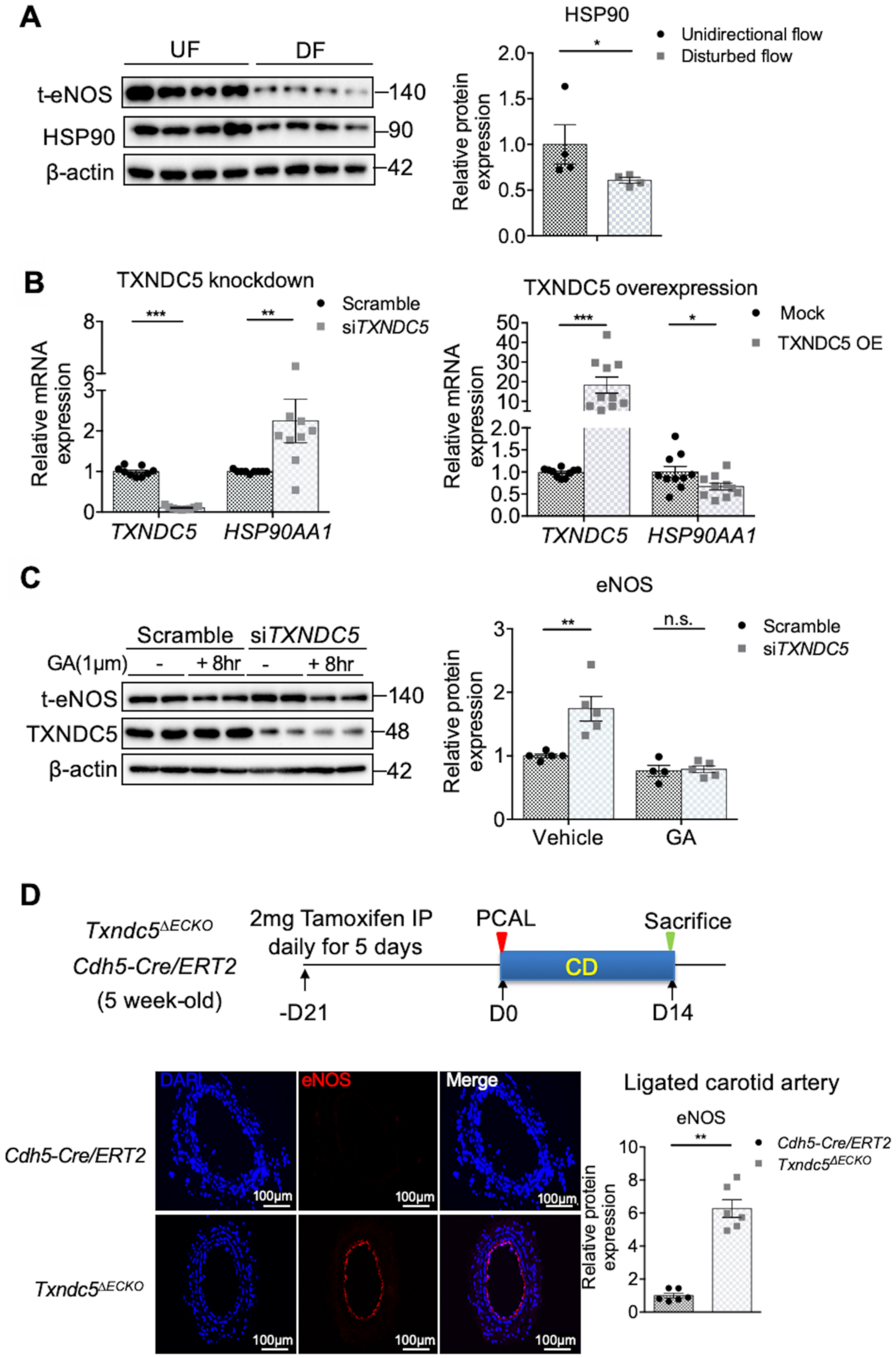

Figure S6. TXNDC5 downregulates eNOS by transcriptional regulation of HSP90.

(A) HSP90 protein expression was reduced in DF-exposed, compared to UF-exposed, HAEC (n=4). **(B)** *TXNDC5* knockdown increased, whereas *TXNDC5* overexpression decreased, *HSP90AA1* mRNA in HAEC (n=9-11). **(C)** Pharmacological inhibition of HSP90 with geldanamycin (GA, 1 μmol/L) abolished TXNDC5 depletion-induced eNOS upregulation in HAEC (n=4-5). **(D)** Endothelium-specific deletion of *Txndc5* by *Cdh5-Cre/ERT2* restored eNOS expression (intensity measured along the vessel lumen) in the ligated LCA in $Txndc5^{\Delta ECKO}$ mice (n=6). (* denotes $p<0.05$, ** denotes $p<0.01$, *** denotes $p<0.001$, n.s.=non-significant determined using two-tailed Mann-Whitney U test).

Figure S7

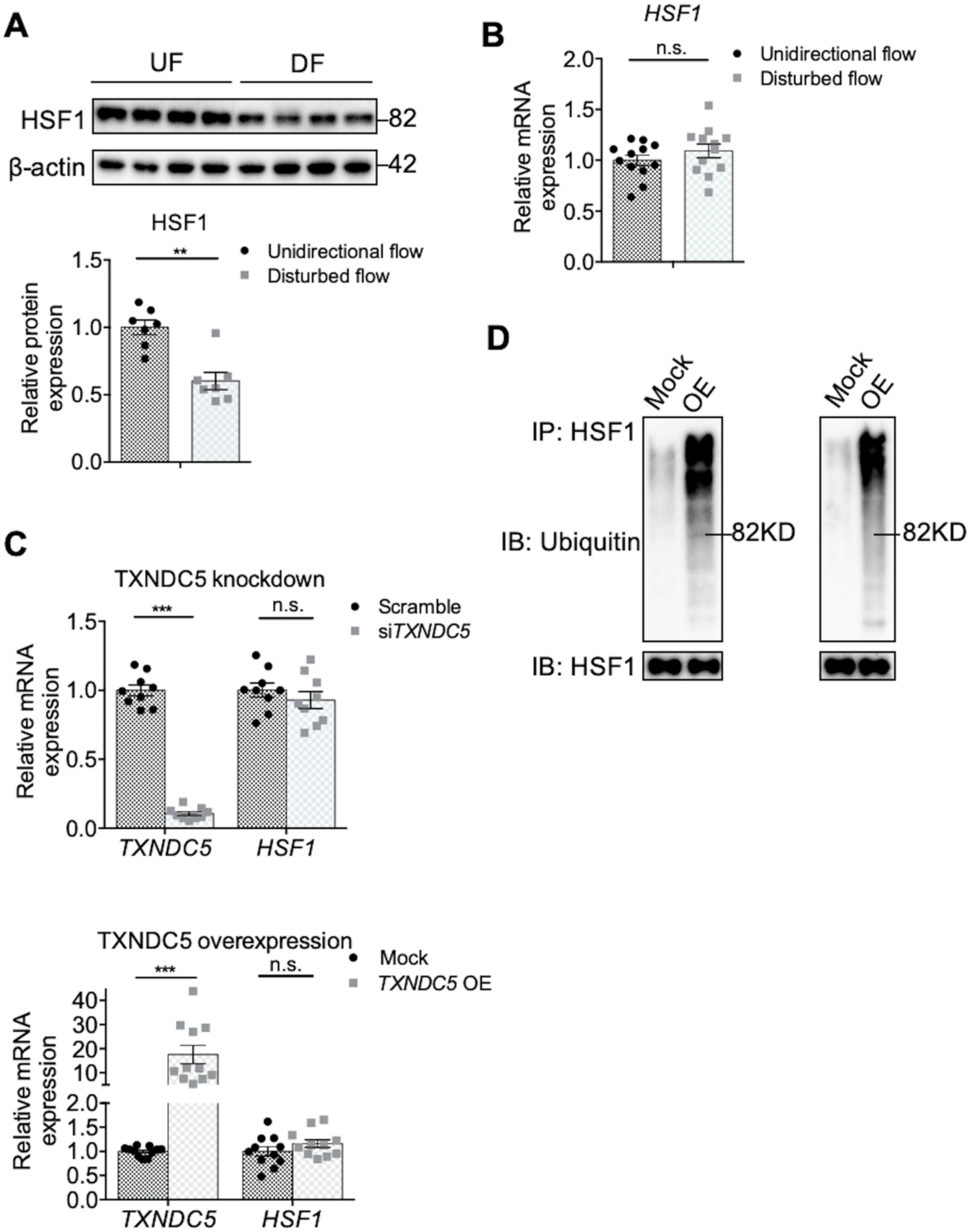

Figure S7. TXNDC5 increases ubiquitin-dependent HSF1 degradation without affecting its transcripts. (A) HSF1 protein expression was reduced in DF-exposed, compared to UF-exposed, HAEC (n=7). **(B)** Endothelial HSF1 transcript level was unaffected by athero-relevant hemodynamic forces (n=12). **(C)** Neither knockdown nor overexpression of *TXNDC5* altered *HSF1* transcript level in HAEC (n=9-11). **(D)** Immunoblots of HSF1 pulled-down protein lysates showed markedly increased ubiquitination of HSF1 in TXNDC5-overexpressed, compared to mock treated, HAEC

(n=2). (** denotes $p<0.01$, *** denotes $p<0.001$, n.s.=non-significant determined using two-tailed Mann-Whitney U test).

Figure S8

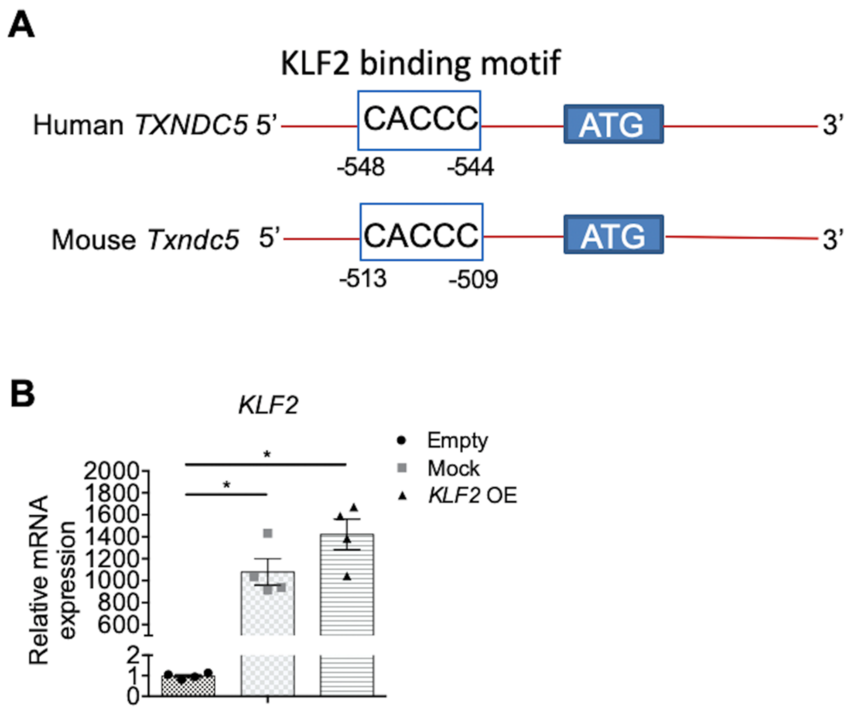

Figure S8. Efficiency of KLF2 overexpression and its binding sites at TXNDC5 promoter region. (A) Scheme of the possible KLF2 binding site at human and mouse TXNDC5 promoter region. **(B)** Robust overexpression of *KLF2* was achieved in *KLF2* wild-type transcript (*KLF2* OE) and untranslated *KLF2* transcript (Mock) compared to empty control (n=4) (* denotes $p<0.05$ using two-tailed Mann-Whitney U test).

Figure S9

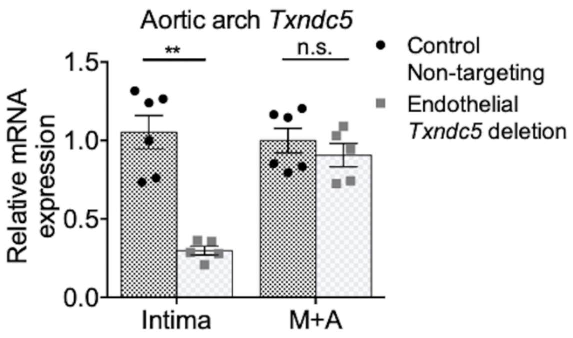

Figure S9. Efficient and specific deletion of endothelial TXNDC5 by nanoparticle-delivered, CRISPR-Cas9-mediated endothelial genome editing *in vivo*. *Txndc5* was efficiently and specifically depleted in the endothelium-enriched intima without affecting the *Txndc5* level in the media and adventitia (M+A), of aortic arch from *ApoE*$^{-/-}$ mice 9 days after single injection of endothelial *Txndc5* deletion nanoparticles (n=5-6). The formulation of the nanoparticles carrying *CDH5*-driven CRISPR/Cas9 is described in Figure 7A. (** denotes p<0.01, n.s.=non-significant determined using two-tailed Mann-Whitney U test).

Supplemental Table 1A. List of upregulated genes in *TXNDC5*-depleted HAEC exposed to disturbed flow

gene_id	gene	locus	value si*TXNDC5*	value siScr	log2(fold_change of si*TXNDC5* vs siScr)	p_value	adjusted p_value
XLOC_000179	LINC01647	1:11609530-11613355	0	1.19788	6.916333323	5.00E-05	0.00963214
XLOC_000496	FAM167B	1:32247232-32248856	3.58526	8.28003	1.20756	5.00E-05	0.00963214
XLOC_001191	KIAA1324	1:109105950-109206781	0.212819	0.830321	1.96404	5.00E-05	0.00963214
XLOC_002198	ATF3	1:212565333-212620777	0.630432	3.48063	2.46493	5.00E-05	0.00963214
XLOC_006223	BAG3	10:119651356-11967789[illegible]	17.9119	28.6605	0.678146	5.00E-05	0.00963214
XLOC_009054	CDKN1C	11:2883112-2885773	0.956324	2.70236	1.49865	5.00E-05	0.00963214
XLOC_009206	NRIP3	11:8980575-9004049	0.815157	2.04501	1.32696	5.00E-05	0.00963214
XLOC_010250	CRYAB	11:111908564-11192691[illegible]	0.566502	24.8451	5.45474	5.00E-05	0.00963214
XLOC_011110	NR4A1	12:52022831-52059507	0.754101	5.83027	2.95073	5.00E-05	0.00963214
XLOC_011756	HSPB8	12:119178641-11922113	3.90747	9.53192	1.28653	5.00E-05	0.00963214
XLOC_012049	CD163,CD163L1	12:7346684-7503893	0.678667	1.92942	1.50739	5.00E-05	0.00963214
XLOC_012659	B4GALNT1	12:57610179-57633355	0.0390696	1.3184	5.0766	5.00E-05	0.00963214
XLOC_012783	PTPRR	12:70638072-70920843	0.203497	0.717612	1.8182	5.00E-05	0.00963214
XLOC_014970	ABHD4	14:22595807-22613215	7.38024	13.2885	0.848443	5.00E-05	0.00963214
XLOC_016034	NFKBIA	14:35401508-35404749	55.301	91.8088	0.731326	5.00E-05	0.00963214
XLOC_017244	MAP1A	15:43510957-43531620	0.0996314	1.63826	4.03942	5.00E-05	0.00963214
XLOC_017472	RNU5B-1	15:65304676-65305408	3897.46	8113.16	1.05773	5.00E-05	0.00963214
XLOC_018948	SOX8	16:981807-986979	0.140339	0.869555	2.63136	5.00E-05	0.00963214
XLOC_018989	SLC9A3R2	16:2025355-2039026	30.9818	51.7971	0.74145	5.00E-05	0.00963214
XLOC_019666	MT1L	16:56617475-56618818	26.1977	55.8398	1.09185	5.00E-05	0.00963214
XLOC_019668	MT1M	16:56632232-56633986	1.14724	4.57448	1.99544	5.00E-05	0.00963214
XLOC_019676	MT1X	16:56682423-56688052	12.3396	69.1965	2.4874	5.00E-05	0.00963214
XLOC_020500	AC138894.1,CLN3,NPIPB7	16:28456370-28498970	11.8899	18.4922	0.637179	5.00E-05	0.00963214
XLOC_021806	CCL2	17:34255217-34257203	37.2952	76.1061	1.02902	5.00E-05	0.00963214
XLOC_022902	AC007952.4	17:19111999-19112636	63.0247	205.671	1.70635	5.00E-05	0.00963214
XLOC_022907	SNORD3C	17:19189664-19190245	167.601	449.675	1.42385	5.00E-05	0.00963214
XLOC_025367	TNFSF9	19:6530998-6535928	0.336805	2.5847	2.94002	5.00E-05	0.00963214
XLOC_025612	KLF2	19:16324816-16327874	3.32981	8.00567	1.26558	5.00E-05	0.00963214
XLOC_026007	PSMC4	19:39971004-39981441	36.9749	63.2873	0.77537	5.00E-05	0.00963214

XLOC_026043	AC243960.3,LINC01480	19:41530221-41536904	1.38799	3.46172	1.31849	5.00E-05	0.00963214
XLOC_026873	DNAJB1	19:14514740-14565980	22.7243	87.6835	1.94807	5.00E-05	0.00963214
XLOC_026942	PLVAP	19:17351447-17377350	3.77191	7.12098	0.916783	5.00E-05	0.00963214
XLOC_031461	SERPINE2	2:223975111-224039319	6.00101	14.5734	1.28006	5.00E-05	0.00963214
XLOC_031880	HSPA12B	20:3732666-3753111	0.334473	0.918059	1.4567	5.00E-05	0.00963214
XLOC_031886	FTLP3	20:4023916-4024444	7.39666	19.2255	1.37808	5.00E-05	0.00963214
XLOC_032430	SLCO4A1	20:62640718-62685785	0.704895	1.59703	1.17991	5.00E-05	0.00963214
XLOC_032483	AL121758.1,SCRT2,SRXN1	20:646614-676179	17.0523	29.5087	0.791173	5.00E-05	0.00963214
XLOC_032622	BFSP1	20:17493904-17569220	1.12153	2.70511	1.27022	5.00E-05	0.00963214
XLOC_033456	MX1	21:41420303-41459214	1.87141	7.49155	2.00114	5.00E-05	0.00963214
XLOC_035332	RN7SL4P	3:15667179-15859771	1745.77	3478.76	0.994711	5.00E-05	0.00963214
XLOC_037443	SLC12A8	3:125082635-125212864	0.106277	0.865327	3.02541	5.00E-05	0.00963214
XLOC_038516	UCHL1	4:41220073-41268459	99.1276	227.249	1.19691	5.00E-05	0.00963214
XLOC_038742	CXCL8	4:73740505-73743716	8.11521	20.0094	1.30198	5.00E-05	0.00963214
XLOC_039018	AC110079.1	4:118591772-118633729	0.123372	2.073	4.07063	5.00E-05	0.00963214
XLOC_041864	GPX3	5:151020437-151029135	5.60346	18.1567	1.69612	5.00E-05	0.00963214
XLOC_042820	LUCAT1	5:90515477-91314547	0.714682	1.68165	1.2345	5.00E-05	0.00963214
XLOC_043689	RNA5SP202	6:4304941-4583871	6301.63	17297.3	1.45675	5.00E-05	0.00963214
XLOC_044051	HSPA1A	6:31815463-31817946	11.0548	85.7738	2.95586	5.00E-05	0.00963214
XLOC_048041	ZFAND2A	7:1151113-1166146	12.1917	41.2641	1.75899	5.00E-05	0.00963214
XLOC_049184	AC074386.1,OR2A1-AS1, OR2A20P	7:144186082-144380632	0.453403	1.11304	1.29563	5.00E-05	0.00963214
XLOC_049203	RNY1	7:148987135-148987248	28526.3	61748.7	1.11412	5.00E-05	0.00963214
XLOC_050922	PLAT	8:42175181-42207724	9.73907	22.8691	1.23154	5.00E-05	0.00963214
XLOC_051302	GEM	8:94249252-94262350	0.431491	1.84187	2.09377	5.00E-05	0.00963214
XLOC_052892	GLDC	9:6532463-6645783	4.64637	8.91798	0.940613	5.00E-05	0.00963214
XLOC_054594	TSPYL2	X:53082309-53088540	5.39865	11.6191	1.10583	5.00E-05	0.00963214
XLOC_003859	RNVU1-18	1:143729406-143729570	280.315	506.083	0.852323	0.0001	0.0172885
XLOC_038745	CXCL1	4:73869392-73871242	18.0865	34.1947	0.918859	0.0001	0.0172885
XLOC_007770	HTATIP2	11:20363684-20383783	15.6431	27.6901	0.823844	0.00015	0.0243705
XLOC_014230	HSPH1	13:31134973-31162388	44.1654	72.8121	0.721262	0.00015	0.0243705
XLOC_016186	LINC00520	14:55780928-55796731	10.0139	25.8226	1.36663	0.00015	0.0243705

XLOC_026183	ZNF114	19:48262899-48287608	0.318365	0.882914	1.47159	0.0002	0.0303034
XLOC_040268	AC078850.1	4:128567971-128570531	0.709102	1.72051	1.27877	0.0002	0.0303034
XLOC_054318	SAT1	X:23783172-23786226	127.972	205.235	0.681454	0.0002	0.0303034
XLOC_016608	CKB	14:103519499-10352311	0.728784	2.30862	1.66347	0.00025	0.0370467
XLOC_012055	GDF3	12:7689781-7695776	1.29613	3.20209	1.3048	0.0003	0.0421406
XLOC_025283	GADD45B	19:2476121-2478456	18.4412	30.6616	0.733498	0.0003	0.0421406
XLOC_051830	RN7SL5P	9:8314245-10612723	4289.33	11204.7	1.38528	0.0003	0.0421406
XLOC_016591	HSP90AA1	14:102080737-10230520[illegible]	151.414	267.374	0.820354	0.00035	0.0471975
XLOC_019155	MIR193BHG	16:14301388-14331067	0.300709	0.658665	1.13118	0.00035	0.0471975
XLOC_027299	BLVRB	19:40447764-40465840	23.0381	37.7022	0.710628	0.00035	0.0471975
XLOC_051839	LURAP1L	9:12685438-12822131	0.548655	1.54415	1.49284	0.00035	0.0471975

Supplemental Table 1B. List of downregulated genes in *TXNDC5*-depleted HAEC exposed to disturbed flow

gene_id	gene	locus	value si*TXNDC5*	value siScr	log2(fold_change of si*TXNDC5* vs siScr)	p_value	adjusted p_value
XLOC_002804	RNU1-1	1:16514121-16514285	8077.57	3621.89	-1.15718	5.00E-05	0.00963214
XLOC_003898	TXNIP	1:145992434-145996600	22.2804	12.2769	-0.859832	5.00E-05	0.00963214
XLOC_005704	CDK1	10:60778330-60794852	15.0188	7.9168	-0.923776	5.00E-05	0.00963214
XLOC_007327	MKI67	10:128096474-12812638[illegible]	8.17741	4.3077	-0.924726	5.00E-05	0.00963214
XLOC_016176	DLGAP5	14:55146517-55191730	13.4747	7.37807	-0.868942	5.00E-05	0.00963214
XLOC_023243	TOP2A	17:40388515-40417950	17.496	8.80025	-0.991406	5.00E-05	0.00963214
XLOC_034479	APOBEC3A,APOBEC3B	22:38952740-38998209	1.34585	0.562919	-1.25752	5.00E-05	0.00963214
XLOC_040003	SCD5	4:82629538-82798857	28.7185	13.558	-1.08284	5.00E-05	0.00963214
XLOC_050764	STC1	8:23841914-23854807	1.7884	0.437684	-2.03071	5.00E-05	0.00963214
XLOC_051337	NIPAL2	8:98189825-98294393	6.94306	2.25818	-1.62041	5.00E-05	0.00963214
XLOC_002215	CENPF	1:214603194-214664588	9.77909	5.49115	-0.832592	0.0001	0.0172885
XLOC_004487	ASPM	1:197084127-197146694	7.20783	4.45853	-0.692995	0.0001	0.0172885
XLOC_006458	FAM107B	10:14517354-14774897	84.4183	52.2723	-0.691509	0.0001	0.0172885
XLOC_010449	ETS1	11:128458760-12858755[illegible]	25.111	15.2263	-0.721753	0.0001	0.0172885
XLOC_013605	CCNA1	13:36431519-36442882	1.34612	0.538773	-1.32106	0.0001	0.0172885
XLOC_048398	IGFBP3	7:45912244-45921874	19.0444	11.2535	-0.758988	0.0001	0.0172885
XLOC_005996	KIF11	10:92593285-92655395	5.68413	3.39507	-0.743498	0.0002	0.0303034
XLOC_040530	HMGB2	4:173331694-173335125	53.5305	33.2598	-0.686582	0.0002	0.0303034
XLOC_042577	DEPDC1B	5:60596911-60700190	2.25724	1.16029	-0.960073	0.0002	0.0303034
XLOC_050610	PPP1R3B	8:9136018-9151574	14.0373	9.11337	-0.623209	0.00025	0.0370467
XLOC_022760	AURKB	17:8204732-8210600	5.72891	3.19218	-0.84372	0.0003	0.0421406
XLOC_035400	TGFBR2	3:30606501-30694142	129.731	84.8477	-0.612576	0.0003	0.0421406

Table S2. Primer list

Gene	Primer sequence
**Human**	
HPRT	F: 5' CGTCTTGCTCGAGATGTGATG 3'<br>R: 5' GCACACAGAGGGCTACAATGTG 3'
β-actin	F: 5' TCCCTGGAGAAGAGCTACGA 3'<br>R: 5' AGGAAGGAAGGCTGGAAGAG 3'
GAPDH	F: 5' TGCACCACCAACTGCTTAGC 3'<br>R: 5' GGCATGGACTGTGGTCATGAG 3'
Ubiquitin	F: 5' ATTTAGGGGCGGTTGGCTTT 3'<br>R: 5' TGCATTTTGACCTGTTAGCGG 3'
TXNDC5	F: 5' CGCACAGCAAGCACCTGTA 3'<br>R: 5' GCGCGAAGAACATGACGAAG 3'
NOS3	F: 5' GAACCCATCCTGCCGTCCTT 3'<br>R: 5' CACGCTGTTGAGGTCGTCG 3'
KLF2	F: 5' GCACGCACACAGGTGAGAAG 3'<br>R: 5' ACCAGTCACAGTTTGGGAGGG 3'
HSP90AA1	F: 5' AAGTCTGGGACCAAAGCGTTC 3'<br>R: 5' GTTCCACGACCCATAGGTTCAC 3'
HSF1	F: 5' TCTCACTGGTGCAGTCAAAC 3'<br>R: 5' GGCTATACTTGGGCATGGAAT 3'
**Mouse**	
Hprt	F: 5' TAATCACGACGCTGGGACTG 3'<br>R: 5' GTTGGGCTTACCTCACTGCT 3'
β-Actin	F: 5' GATCAAGATCATTGCTCCTCCTG 3'<br>R: 5' AGGGTGTAAAACGCAGCTCA 3'
Gapdh	F: 5' TGCACCACCAACTGCTTAGC 3'<br>R: 5' GGCATGGACTGTGGTCATGAG 3'
Ubiquitin	F: 5' AGTGACGAGAGGCTTTGTCC 3'<br>R: 5' CGAAGATCTGCATTTTGACCTGT 3'
Txndc5	F: 5' AGGATACCCCACCCTGAAGT 3'<br>R: 5' GCTCAAAGTTGTTGGCCGAG 3'
***Txndc5*_exon2-3**	F: 5' GTGGACTGCACGGCTGATTC 3'<br>R: 5' GCAGCATCCAGTTTTCCAGTG 3'
Nos3	F: 5' GAAGGCGTTTGATCCCCGGGTCCTG 3'<br>R: 5' CAGCTCCTCCAGCCTTGTGTCCAC 3'
Klf2	F: 5' GCGTACACACACAGGTGAGA 3'<br>R: 5' GCACAAGTGGCACTGAAAGG 3'

Hsp90aa1	F: 5’ AATGCTTAGAACTATTTACTGAACTAGCAGAA 3’
	R: 5’ GTCCTCGTGAATTCCAAGCTTT 3’
Pecam-1	F: 5’ AGCCTAGTGTGGAAGCCAAC 3’
	R: 5’ CTGTACACCGTCTCTGTGGC 3’
Acta2	F: 5’ TTTCCAAATCATTCCTGCCC 3’
	R: 5’ CDCTCTCAAATACCCCGTTT 3’

Table S3. Antibody list

Target antigen	Vendor	Cat number	Species	Working concentration
**β-actin**	Abcam, Cambridge, UK	ab6276	Mouse	WB: 1:3000
**CD-11b**	BioLegend, CA, USA	420301	Rat	Flow cytometry 1:100
**phospho-eNOS, Ser 1177**	BD biosciences, NJ, USA	612393	Mouse	WB: 1:1000
**total-eNOS**	Cell Signaling Technology, MA, USA	32027	Rabbit	WB: 1:1000<br>IF: 1:50-100
**HSF-1**	Cell Signaling Technology, MA, USA	4356	Rabbit	WB: 1:1000
**HSP90**	BD Biosciences, NJ, USA	610418	Mouse	WB: 1:4000
**TXNDC5**	Proteintech, IL, USA	19834-1-AP	Rabbit	WB: 1:50000<br>IF: 1:500-1000
**Ubiquitin**	Cell Signaling Technology, MA, USA	3936	Mouse	WB: 1:1000
**VE-Cadherin**	BD Biosciences, NJ, USA	555289	Rat	IF: 1:50-100

IF: immunofluorescence, IHC: immunohistochemistry, WB: western blotting
